# Neuroinflammatory disease signatures in *SPG11*-related hereditary spastic paraplegia patients

**DOI:** 10.1007/s00401-023-02675-w

**Published:** 2024-02-02

**Authors:** Laura Krumm, Tatyana Pozner, Naime Zagha, Roland Coras, Philipp Arnold, Thanos Tsaktanis, Kathryn Scherpelz, Marie Y. Davis, Johanna Kaindl, Iris Stolzer, Patrick Süß, Mukhran Khundadze, Christian A. Hübner, Markus J. Riemenschneider, Jonathan Baets, Claudia Günther, Suman Jayadev, Veit Rothhammer, Florian Krach, Jürgen Winkler, Beate Winner, Martin Regensburger

**Affiliations:** 1 Department of Stem Cell Biology, Friedrich-Alexander-Universität (FAU) Erlangen-Nürnberg, Erlangen, Germany; 2 Department of Neuropathology, FAU Erlangen-Nürnberg, Erlangen, Germany; 3 Institute of Functional and Clinical Anatomy, FAU Erlangen-Nürnberg, Erlangen, Germany; 4 Department of Neurology, University Hospital Erlangen, FAU Erlangen-Nürnberg, Erlangen, Germany; 5 Division of Neuropathology, Department of Laboratory Medicine and Pathology, University of Washington, Seattle, WA USA; 6 Department of Neurology, University of Washington Medical Center, Seattle, WA USA; 7 VA Puget Sound Healthcare System, Seattle, WA USA; 8 Department of Medicine 1, University Hospital Erlangen, FAU Erlangen-Nürnberg, Erlangen, Germany; 9 Institute of Human Genetics, Jena University Hospital Friedrich-Schiller-University Jena, Jena, Germany; 10 Center for Rare Diseases, Jena University Hospital, Friedrich Schiller University Jena, Jena, Germany; 11 Department of Neuropathology, Regensburg University Hospital, Regensburg, Germany; 12 Translational Neurosciences, Faculty of Medicine and Health Sciences, University of Antwerp, Antwerp, Belgium; 13 Laboratory of Neuromuscular Pathology, Institute Born-Bunge, University of Antwerp, Antwerp, Belgium; 14 Neuromuscular Reference Centre, Department of Neurology, Antwerp University Hospital, Antwerp, Belgium; 15 Deutsches Zentrum Immuntherapie (DZI), University Hospital Erlangen, Kussmaulallee 4, 91054 Erlangen, Germany; 16 Institute for Stem Cell and Regenerative Medicine, University of Washington, Seattle, WA USA; 17 Division of Medical Genetics, University of Washington, Seattle, WA USA; 18 Center for Rare Diseases Erlangen (ZSEER), University Hospital Erlangen, FAU Erlangen-Nürnberg, Erlangen, Germany; 19 Department of Molecular Neurology, FAU Erlangen-Nürnberg, Erlangen, Germany

**Keywords:** Multisystem neurodegeneration, Inflammation, IFNγ/ STAT1 signaling, Autosomal-recessive hereditary spastic paraplegia, Induced microglia-like cells, Disease-associated microglia

## Abstract

Biallelic loss of *SPG11* function constitutes the most frequent cause of complicated autosomal recessive hereditary spastic paraplegia (HSP) with thin corpus callosum, resulting in progressive multisystem neurodegeneration. While the impact of neuroinflammation is an emerging and potentially treatable aspect in neurodegenerative diseases and leukodystrophies, the role of immune cells in SPG11–HSP patients is unknown. Here, we performed a comprehensive immunological characterization of SPG11–HSP, including examination of three human *postmortem* brain donations, immunophenotyping of patients’ peripheral blood cells and patient-specific induced pluripotent stem cell-derived microglia-like cells (iMGL). We delineate a previously unknown role of innate immunity in SPG11–HSP. Neuropathological analysis of SPG11–HSP patient brain tissue revealed profound microgliosis in areas of neurodegeneration, downregulation of homeostatic microglial markers and cell-intrinsic accumulation of lipids and lipofuscin in IBA1^+^ cells. In a larger cohort of SPG11–HSP patients, the ratio of peripheral classical and intermediate monocytes was increased, along with increased serum levels of IL-6 that correlated with disease severity. Stimulation of patient-specific iMGLs with IFNγ led to increased phagocytic activity compared to control iMGL as well as increased upregulation and release of proinflammatory cytokines and chemokines, such as CXCL10. On a molecular basis, we identified increased STAT1 phosphorylation as mechanism connecting IFNγ-mediated immune hyperactivation and *SPG11* loss of function. STAT1 expression was increased both in human *postmortem* brain tissue and in an *Spg11*^*–/–*^ mouse model. Application of an STAT1 inhibitor decreased CXCL10 production in SPG11 iMGL and rescued their toxic effect on SPG11 neurons. Our data establish neuroinflammation as a novel disease mechanism in SPG11–HSP patients and constitute the first description of myeloid cell/ microglia activation in human SPG11–HSP. IFNγ/ STAT1-mediated neurotoxic effects of hyperreactive microglia upon *SPG11* loss of function indicate that immunomodulation strategies may slow down disease progression.

**Supplementary Information:**

The online version contains supplementary material available at 10.1007/s00401-023-02675-w.

## Introduction

Hereditary spastic paraplegias (HSP) are a heterogeneous group of genetic motor neuron disorders, characterized by progressive spasticity and weakness of the lower limbs due to degeneration of corticospinal motor neurons and ascending dorsal columns [8, 54, 56, 70]. The most frequent form of autosomal-recessive complicated HSP is caused by pathogenic bi-allelic variants in the *SPG11* gene leading to a loss of *SPG11* function [56]. In addition to spastic paraparesis, *SPG11*-related HSP (SPG11–HSP) is commonly characterized by cognitive impairment, thin corpus callosum, and amyotrophy [56]. While the precise function of *SPG11* encoding spatacsin is not fully understood, its loss is suggested to be involved in failure of the autophagic lysosomal reformation, which results in accumulation of autophagosomes and depletion of lysosomes [15, 18, 46, 99]. In addition, loss of spatacsin leads to impaired cholesterol trafficking and progressive ganglioside deposition in neurons [13, 14]. In patient-derived neurons from induced pluripotent stem cells (iPSCs), we have previously demonstrated neurodevelopmental deficits as well as impaired neurite complexity and transport defects of SPG11 neurons [34, 60, 71, 72, 77]. For other motor neuron disorders and neurodegenerative diseases, including Alzheimer’s disease (AD), Parkinson’s disease (PD), and amyotrophic lateral sclerosis (ALS), it is well-established that neuroinflammation contributes to neuronal loss [35, 80, 108]. These neuroinflammatory processes involve brain resident myeloid cells including microglia exerting host defense against pathogens and maintaining brain homeostasis [78, 80]. In an increasing number of neurodegenerative disorders, specific microglia subtypes with distinct transcriptomic signatures including disease-associated microglia (DAM) are considered to impact pathogenicity.

Neuroinflammatory changes, characterized by brain-infiltrating CD8^+^ T cells, amplified neurodegeneration in a recently published *Spg11*^–/–^ mouse model [41]. The exact mechanism of CD8^+^ T-cell invasion in *Spg11*^–/–^ mice remained unclear. Activation of microglia, however, was observed in different brain regions, which could potentially initiate enhanced recruitment of pathogenic CD8^+^ T cells. *SPG11* mRNA is highly expressed in human PBMCs and lymphoblasts [37], and levels of proinflammatory resistin were increased in SPG11–HSP patients which adds further indications for a role of innate inflammation in SPG11–HSP [81]. In this study, we hypothesized that altered myeloid cell states are an important disease mechanism in SPG11–HSP. Evaluation of SPG11–HSP *postmortem* brain tissues revealed severe and widespread microgliosis and disease-associated microglia (DAM) marker expression. Elevated blood serum levels of proinflammatory monocytes and the proinflammatory cytokine IL-6 were detected in an SPG11 patient cohort. Finally, stimulation by IFNγ triggered a hyperactivated state in patient-derived induced microglia-like cells (iMGL) including increases in phagocytosis and in expression and secretion of proinflammatory cytokines and chemokines via a STAT1-dependent mechanism. Increased STAT1 signaling was confirmed in the SPG11 *postmortem* case as well as an *Spg11*^–/–^ mouse model. Moreover, conditioned media of IFNγ treated SPG11 iMGL induced neuronal cell death and this effect was prevented by inhibition of STAT1.

## Materials and methods

### Patients

This study was approved by the local Institutional Review Board of the Friedrich-Alexander-Universität Erlangen-Nürnberg (No. 17-259-B, No. 17-347-B, No. 21-498-D, No. 484_20 B), and all individuals provided written informed consent. Brain sampling followed the ethical rules of each country.

### *Postmortem* tissue

“SPG11 (UKER)” *postmortem* tissue was donated by a female SPG11–HSP patient (SPG11 donor SPG11-3 in the iMGL experiments, Online Resource Table 3) with a disease onset at the age of 31, progressive gait disorder, muscle cramps, and loss of dexterity. Genetic testing identified a heterozygous nonsense variant at c.267G > A/p.Trp89X in exon 2 and the splice site variant c.1457-2A > G in intron 6 of *SPG11*. During disease progression, the patient developed a spastic paraparesis, muscle cramps, distal muscle wasting, and pseudobulbar dysarthria. At age 46, she had lost ambulation and exhibited progressive dementia and dysphagia. The patient died at the age of 51 of aspiration, without clinical evidence of an inflammatory disorder, and without antibiotic or immunomodulatory therapy. *Postmortem* brain tissue derived from a female without neurological disease deceased at age 42 from arterial lung embolism was used as non-inflammatory “control”.

The “SPG11 (BG2)” case was previously described in Denora et al. 2016 [25]. Briefly, this *postmortem* tissue is derived from a female SPG11–HSP patient with disease onset at age 10, rapidly progressing spastic cerebellar ataxic gait and mild intellectual disability. The patient died at the age of 46 years, and familial genetic testing revealed a homozygous truncating variant c.6739_6742del (p.Glu2247Leufs14) in exon 36 of *SPG11*. Corresponding control tissue derived from a female deceased at age 59 from the same center was used.

The “SPG11 (UWA)” *postmortem* tissue was donated by a male SPG11–HSP patient from the Genetic Medicine Clinic at University Washington Medical Center, USA, who died at the age of 42. This patient had delayed language acquisition as a child and required special education through high school. He presented at age 20 with gait and balance symptoms that had begun a few years earlier. Examination showed nystagmus, distal upper extremity amyotrophy and spasticity. He was diagnosed with optic atrophy at age 34. By the time of death, he was mute and markedly bradykinetic. Genetic testing in this case identified a homozygous 4 base-pair deletion variant c.6439_6442del leading to a frameshift. A detailed clinical workup of this case will be published elsewhere (Scherpelz et al., in review). Corresponding control tissues derived from two males deceased without neurological diseases at age 38 (control UWA1) and at age 42 (control UWA2) from the same center were used.

Each SPG11 *postmortem* case was compared only with corresponding control tissue subjected to comparable processing and staining procedures at the same facility.

### Blood samples

Peripheral blood samples were collected from SPG11–HSP patients and healthy controls (Online Resource Tables 1 and 2). All individuals were asked to avoid excessive physical activity within the preceding week, and blood samples were withdrawn in the morning (between 8 and 11 am) after a fasting period of at least 8 h, including abstinence from caffeine and sweeteners. Serum samples were cooled immediately after blood drawing, and supernatants were prepared within 4 h and subsequently stored at  – 80 °C [81]. Individuals receiving immunomodulatory medications were not included in the study. Peripheral blood mononuclear cells (PBMCs) were obtained from patients with non-neurodegenerative or non-inflammatory diseases (controls, *n* = 38) and SPG11 patients (*n* = 8). The PBMC control cohort consisted of non-inflammatory and non-degenerative neurological diseases, i.e., meningioma (*n* = 1), idiopathic intracranial hypertension (*n* = 8), dysesthesia (*n* = 7), pain syndrome (*n* = 2), primary headache (migraine, tension headaches; *n* = 6), normal pressure hydrocephalus (*n* = 2), somatization disorder (*n* = 2), polyneuropathy (*n* = 1), fatigue (*n* = 1), phobic vertigo (*n* = 1), non-specific white matter lesions (*n* = 4) and without a diagnosis (*n* = 3). PBMCs underwent processing using a Ficoll gradient method and were subsequently preserved in liquid nitrogen (Ficoll–Paque PLUS density gradient media, Cytiva) [84]. Viability rates exceeded 80%, and routine quality assessments showed unchanged phenotypes of frozen PBMCs compared to the analysis of fresh PBMCs [12, 84].

### iPSC lines

IMGL were differentiated from iPSCs derived from three Caucasian females with compound heterozygous pathogenic variants in the *SPG11* gene, including the “SPG11 (UKER)” *postmortem* case, and three age- and sex-matched healthy controls (Online Resource Table 3). Their precise clinical phenotype and generation of iPSCs have been described previously [5, 39, 72, 90].

### Animals

Generation and characterization of *Spg11*–KO mice was described previously [46, 99]. All animal experiments were approved by the “Thüringer Landesamt für Lebensmittelsicherheit und Verbraucherschutz” (TLLV) in Germany (Approval number: 02–039-14). Mice were housed in a 12 h light/dark cycle and fed on a regular diet ad libitum. Five *Spg11*^+/+^ and five *Spg11*^−/−^ mice were analyzed at the age of 16 months.

### MSD® multi-spot assay system

Multiplex electrochemiluminescence (ECL) was performed using the Meso Scale Discovery® system (MSD®; Rockville, MD, USA) according to the manufacturer’s instructions, using the U-PLEX Custom Biomarker Group 1 (human) Assay (Cat.: K15067L-1), including U-PLEX 10-Assay, 96-Well SECTOR Plate (Cat.: N05235A-1), U-PLEX Human Antibody Sets for IFNγ (Cat.: B21TT-2), IL-1β (Cat.: B21TU-2), IL-6 (Cat.: B21TX-2), IL-8 (Cat.: B21TY-2), IL-10 (Cat.: B21TZ-2), TNFα (Cat.: B21UC-2), IP-10 (Cat.: B21UF-2), MCP-1 (Cat.: B21UG-2), IL-1α (Cat.: B21UN-2) and IL-18 (Cat.: B21VJ-2). In addition, calibrator 1 (Cat.: C0060-2), calibrator 2 (Cat.: C0061-2) and calibrator 3 (Cat.: C0062-2) were applied together with the respective buffers, Diluent 43 (Cat.: R50AG) and Diluent 3 (Cat.: R50AP). Patient serum and cell culture supernatants were diluted 1:1 in Diluent 43. Provided plates were coated with Linker-coupled antibodies 1 day before the assay was performed. The biotinylated antibodies were combined with the assigned Linker (Linker 1 for IFN-γ, Linker 2 for IL-1β, Linker 3 for IL-6, Linker 4 for IL-8, Linker 5 for IL-10, Linker 6 for TNF-α, Linker 7 for IP-10, Linker 8 for MCP-1, Linker 9 for IL-1α and Linker 10 for IL-18) and adjusted to 6 ml with the Stop Solution (Cat.: R50AO-1). 50 µl of the coating solution was added to each well and the plates were subsequently incubated for 1 h at room temperature, washed trice with phosphate-buffered saline (PBS)/ 0.05% Tween-20. The plates were stored at 4 °C overnight. The calibrators were diluted in a fourfold serial dilution using Diluent 43 to generate eight standards. 25 µl of Diluent 43 was added to each well, followed by 25 µl of prepared calibrator standards or sample dilutions. All standards and samples were measured in duplicates. The plates were incubated at RT with shaking for 1 h. After the plates were washed three times as described above, 50 µl of detection antibody solution was added to each well. The 100X stock solution of the detection antibodies was diluted in Diluent 3. After an incubation for 1 h, the plates were washed again twice and 150 µl of MSD GOLD Read Buffer B was added to each well. The measurement was performed on MESO® QuickPlex® SQ 120MM (Cat.: Al1AA) and subsequent analysis were carried out using MSD® Discovery Workbench® Version 4.0. Protein concentrations within the iMGL supernatants were further normalized to total protein amount, which was measured using the Pierce BCA protein assay Kit (Thermo Fisher) according to the manufacturer’s protocol.

### Immunohistochemistry (IHC) of human *postmortem* tissue

Formalin-fixed paraffin-embedded tissue blocks of cortical areas and basal ganglia sectioned at 5 µm underwent standard analyses, including Hematoxylin and Eosin (H&E) and luxol fast blue (LFB) staining. Chromogenic (DAB) immunohistochemistry was performed for GFAP, CD8, CD4, CD68, STAT1 and IBA1. For immunofluorescence (IF) stainings, sections were deparaffinized at 60 °C for 30 min and incubated in Neo-Clear (Sigma-Aldrich) for 10 min. After rehydration, antigen retrieval was performed by incubating sections in 100X Tris Buffer (pH 10.0; abcam) using a pressure cooker (2100 Antigen Retriever, Aptum Biologics). Sections were permeabilized for 10 min with 0.2% Triton-X-100 (Sigma-Aldrich) and 2% bovine serum albumin (BSA) in PBS followed by blocking of non-specific antibody binding by PBS with 2% BSA for 1 h at RT. Primary antibody incubation in PBS 2% BSA overnight at 4 °C (Online Resource Table 4). On the next day, sections were first blocked with 2% donkey serum (Pan Biotech) and 0.2% Triton-X-100 in PBS and afterward incubated in respective secondary Alexa Fluor coupled donkey antibody (1:500, Thermo Fischer, Online Resource Table 4) in PBS 2% BSA for 1 h at RT. Next, sections were washed in PBS with 0.2% Triton-X-100 for 5 min followed by PBS washes. In addition, sections were then stained with 0.5 mg/ml 49,6-diamidino-2-phenylindole (DAPI) and autofluorescence was quenched with TrueBlack (20X in 70% Ethanol, Biotium). Sections were subsequently washed and mounted with Prolong Gold anti-fade with DAPI Mounting Media (Invitrogen). Microscopic analysis was performed on an Observer.Z1 microscope (ZEISS) and ZEN 2.6 blue software (ZEISS). For the quantification of the IF images, ten randomly selected fields of view were counted manually, blinded for genotype for each brain region.

### IHC of murine brain tissue

Animals were euthanized with a fivefold overdose of Ketamin (500 mg/kg body weight) and Xylazin (80 mg/kg body weight) and perfused transcardially with 4% PFA in 1 × PBS. Brains were removed and post-fixed in 4% PFA overnight at 4 °C.

IHC was performed on 40 µm cryosections that were first washed three times with TBS buffer (100 mM TRIS, 1.5 M NaCl, pH 8 in H_2_O) for 5 min. Afterward, antigen retrieval was performed. Sections were incubated in Dako TRS, Citrate pH 6 (10x; Aglilent Technologies) for 30 min at 80 °C followed by 30 min at RT. After washing with TBS, sections were incubated in blocking buffer (3% donkey serum, 0.1% Triton-X-100 in TBS) for 30 min. Incubation of the primary antibody (Online Resource Table 4) diluted in blocking buffer was performed at 4 °C overnight. On the next day, sections were first washed with TBS and afterward incubated in respective secondary Alexa Fluor coupled donkey antibody (Online Resource Table 4) for 1 h at RT. Sections were subsequently stained with 0.5 mg/ml DAPI and washed with TBS. Mounting was performed using Prolong Gold anti-fade Mounting Media (Invitrogen). Microscopic analysis was performed as described above. For quantification of the IF images, five randomly selected fields of view per mouse were counted manually, blinded for genotype.

### Electron microscopy

*Postmortem* brain tissue fixed in 4% PFA underwent two different routes for further processing. For ultrastructural analysis, tissue was post-fixed in Itho-buffer containing 3% glutaraldehyde (GA). Embedding in resin and sectioning was performed as described before [1]. For immuno-gold labeling, tissue samples were rehydrated, frozen and cryo-sectioned to 50 µm. These 50 µm sections were then incubated with an anti-IBA1 antibody (Wako, Online Resource Table 4) and a gold-labeled secondary antibody. After washing the section to remove unbound secondary antibodies, silver enhancement was performed to increase signal intensity in transmission electron microscopic (TEM) analysis. Sections were then fixed in Itho-buffer (with 3% GA), embedded and sectioned as described above. All ultrathin sections were transferred into a 1400Plus TEM (JEOL) operating at 120 kV.

### iPSC derivation and culture

Fibroblasts from patients and controls had been reprogrammed as previously described [72, 76]. Briefly, fibroblasts obtained from dermal punch biopsies were reprogrammed with viral transduction [34, 72] of the four Yamanaka factors (KLF4, c-Myc, Oct4 and Sox2). All obtained iPSC lines were tested for pluripotency (Tra1-60 expression by Flow cytometry) and stable karyotype using the G-banding chromosomal analysis and analysis of copy number variations > 100 kb [76]. All iPSC lines were described in our previous studies which focused on the neural lineage [34, 51, 60, 71, 72, 77]. Two established iPSC lines were analyzed per individual.

iPSCs were maintained in mTeSR Plus (STEMCELL Technologies) on Geltrex™-coated plates (500 µg for 57 cm^2^, Thermo Fisher Scientific). Cells were passaged as clumps using Gentle Cell Dissociation reagent (STEMCELL Technologies).

### iMGL generation

iPSCs were differentiated into iMGL via hematopoietic progenitor cells (HPCs) as previously described [52]. For HPC generation, STEMDiff hematopoietic kit (STEMCELL Technologies) was used according to the manufacturer’s protocol. Briefly, iPSCs were seeded as small clumps and cultivated in the media provided by the STEMDiff hematopoietic kit. HPCs were collected on days 12, 14, and 16 of differentiation and combined in RPMI1640 with 10% FCS (Gibco), 50 U/ml Penicillin/streptomycin (Gibco), and 10 ng/ml GM–CSF (Peprotech). On day 16 of differentiation, HPCs were either frozen or further seeded in maturation media additionally containing 50 ng/ml IL-34 (Peprotech) on glass slides containing plates to generate iMGL. HPCs were differentiated for 2 weeks into iMGL with the addition of media every 2–3 days and one 50% media change after 1 week. All analyses were performed on differentiated iMGL, i.e., after 2 weeks on day 28, including these stimuli: IFNγ (10 ng/ml for 24 h, Peprotech), LPS (100 ng/ml for 24 h, SIGMA), Oleic Acid (200 µm for 24 h; SIGMA), serum depletion for 24 h with BafilomycinA1 (BAF) exposure (100 nM for 6 h; Thermo Fisher) and Ruxolitinib (5–100 µM for 24 h before IFNγ stimulation, in DMSO, Selleckchem). For immunocytochemical analysis, either 100,000 HPCs per well were differentiated in a 24-well plate or differentiated iMGLs were reseeded with TrypLE Express (Invitrogen) on a 96-well staining plate (Ibidi; 10,000 cells/ well).

To generate microglia-conditioned media (MCM), control and SPG11 iMGL (5 clones each) were re-seeded on day 25 of differentiation on 24-well plates with 200,000 cells per well. On day 27, Ruxolutinib treatment (50 µM, Selleckchem) was performed, 24 h prior to IFNγ (10 ng/ml, Peprotech). After 24h of IFNγ stimulation, MCM was collected and centrifuged at 1000 × g for 10 min. The supernatant was diluted 1:1 in neuronal media NMM and applied to neurons.

### Generation of iPSC-derived neurons

Differentiation of iPSC into cortical neurons was based on the published protocol by Shi et al. [86]. First, iPSCs were seeded at a density of 300,000 cells/cm^2^ on Geltrex™-coated plates. At 100% confluency, medium was changed to neural induction medium which is composed of neural maintenance medium (NMM; 50% Neurobasal (Life Technologies), 50% DMEM/F12 + GlutaMAX (Life Technologies), B27 + VitA (50x; Life Technologies), N2 (200x; Life Technologies), MEM Non-essential amino acids solution (100x, Life Technologies), 2-Mercaptoethanol (50 µM; Life Technologies), Penicillin/streptomycin (50 U^*^ml^−1^; Life Technologies)) supplemented with LDN (100 nM; Tocris) and SB431542 (10µM; Tocris). Medium was changed every day until day 12 when the cells were split 1:3 in aggregates using Collagenase IV (Life Technologies) and medium was changed to NMM with subsequent media changes every other day. As soon as neural rosettes appeared, NMM was supplemented with FGF2 (20 ng/ml; Peprotech) for 2 days. After withdrawal of FGF2, approximately 16–20 days after neural induction, cells were split again 1:2 using Collagenase IV. On day 27 of differentiation, neural progenitor cells (NPCs) were detached by Accutase (Life Technologies) and cryopreserved. For further differentiation into neurons, NPCs were seeded at a density of 50,000 cells/cm^2^ on Geltrex™-coated plates and cultured in NMM with half media changes twice weekly. For conditioned media experiments, neurons were split after 2 weeks of differentiation on 48-well plates with 300,000 cells per well. After 48 h of incubation with MCM, neurons were fixed for 10 min in 4% PFA in PBS, followed by three washes with PBS. As a positive control, neurons were incubated with sodium arsenite (10 nM for 24 h) to induce cell death.

### Phagocytosis assay

To confirm and quantify the phagocytic activity of iMGL, pHrodo™ Red S. aureus BioParticles™ (Thermo Fisher) were used according to the manufacturer’s protocol, as described previously [52]. Shortly, iMGL were dissociated on day 26 of differentiation using TrypLE Express and 20,000 cells/ 96-well were seeded in a fluorescence reader compatible 96-well plate (Corning). After 24 h, iMGL were exposed to IFNγ (10 ng/µl for 24 h) or left untreated and on the next day, the phagocytosis assay was performed. iMGL of each line were additionally treated with CytochalasinD (10 µM; Life Technologies) for 30 min at 37 °C as a negative control, before pHrodo particles were added (200 ng/ml in maturation media). All iMGL were incubated with bacterial particles for 2 h at 37 °C before cell nuclei were counterstained with NucBlue (Thermo Fisher) for 20 min according to the manufacturer’s protocol. Fluorescence intensity was measured in triplicates at 2 h, 4 h, 6 h and 8 h after the addition of pHrodo particles on a CLARIOstar Plus fluorescent plate reading device (BMG LABTECH).

### Immunocytochemistry

Differentiated iMGL and MCM-exposed neurons were fixed for 10 min in 4% PFA in PBS, followed by three washes with PBS. To permeabilize the cells and block unspecific antibody binding, cells were first incubated for 1h in PBS with 3% donkey serum and 0.1% Triton-X-100 at RT. Primary antibodies diluted in PBS with 3% donkey serum were applied (Online Resource Table 5) and incubated overnight at 4 °C or 1 h at RT. Afterward, cells were washed three times with PBS and incubated for 1 h at RT with the respective Alexa Fluor coupled secondary antibody diluted in PBS with 3% donkey serum (Online Resource Table 4).

To visualize lysosomal proteins in iMGL, cells were first incubated in 50 mM NH_4_Cl in PBS for 10 min to reduce the autofluorescence. After washing one time with PBS, cells were permeabilized with 0.1% saponin in PBS for 10 min followed by blocking of non-specific antibody binding with 5% donkey serum and 0.05% saponin in PBS for 1 h. Primary antibodies were diluted in 3% donkey serum and 0.05% saponin in PBS and incubated overnight at 4 °C or 1 h at RT. Next, cells were washed three times with PBS followed by 1 h incubation at RT with secondary antibodies which were diluted in PBS with 3% donkey serum and 0.05% saponin.

After staining with secondary antibodies, nuclei were counterstained with DAPI (0.5 mg/ml) for 5 min followed by three PBS washes. Cells stained on coverslips were mounted using Aqua-Poly/Mount (Polyscience). If staining was performed in a 96-well plate (ibidi), cells were mounted with Mowiol 4–88 (Sigma-Aldrich). Before microscopic analysis, mounted coverslips and plates were dried overnight at RT.

For visualization of apoptotic cells, the One-step TUNEL In Situ Apoptosis Kit (Red, Elab Fluor® 647; Elabscience) was used before antibody staining according to the manufacturer’s instructions.

### Flow cytometry (FC)

For FC analysis of PBMCs, frozen PBMCs were thawed and allowed to rest in a medium at a temperature of 37 °C. Subsequently, centrifugation was carried out at 300 g for 10 min. The PBMCs were then suspended in FACS buffer, which consisted of PBS (PAA Laboratories GmbH) supplemented with 2% fetal calf serum (FCS; Invitrogen). Blocking was performed using human Fc-block (BD Bioscience). PBMCs were stained for 30 min at 4 °C, washed, and finally analyzed using a Cytek Northern Lights instrument (Cytek Biosciences, Fremont, CA, USA). The following antibodies were used for staining: LIVE/DEAD Zombie NIR Fixable Viability Kit (Biolegend), CD3 cFluor® V420 (Cytek, CloneSK7), HLA-DR PerCP (BD, Clone L243), CD14 PerCP eFluor 710-A (Invitrogen, Clone 61D3), CD19 cFluorBYG710-A (Cytek, Clone HIB19), CD16 cFluor R668-A (Cytek, Clone 3G8), CD56 cFluor R720-A (Cytek Clone 5.1H11). A detailed gating strategy is provided in Online Resource Fig. 3.

FC analysis of IBA1 expression in iMGL was performed as described previously [52]. Briefly, iMGL were dissociated and collected in FC buffer (2% FCS and 0.01% sodium azide in PBS) on day 28 of differentiation. After centrifugation, 100,000 cells per staining were transferred in a V-bottom 96-well plate. Cells were fixed by 10 min incubation in 50 µl BD Cytofix per well (BD Bioscience) followed by permeabilization for 5 min in BD Perm/Wash (BD Bioscience). Afterward, cells were incubated for 30 min at RT with primary antibody anti-IBA1 (Wako; Online Resource Table 4) diluted 1:50 in BD Perm/Wash. Cells were washed and secondary antibody incubation for 30 min was performed (Thermo Fisher; Online Resource Table 4) followed by additional washing steps. Finally, cells were resuspended in FC buffer for analysis on a Beckman Coulter Cytoflex S FACS platform. As negative controls, iMGL stained with respective rabbit isotype control (Invitrogen) and iPSC stained for IBA1 were included. Data were analyzed using CytExpert software (version 2.4.0.28).

### Western blot

For protein isolation, cells were pelletized in cold PBS. Pellets were resuspended in 100–150 µl Radio-immunoprecipitation buffer (RIPA; 50 mM Tris/HCl pH 7.4, 0.5% Deoxycholic acid sodium salt, 1% NP-40, 1% Sodium deoxycholate, 0.1% Sodium dodecyl Sulfate (SDS), 150 mM Sodium chloride, 2 mM Ethylenediaminetetraacetic acid (EDTA), 50 mM Sodium fluoride) freshly supplemented with EDTA free complete mini protease inhibitor cocktail (Roche) and PhosphoSTOP (Roche). Cells were incubated for 30 min on ice followed by sonication with Diagenode Bioruptor Pica (setting: 30 s ON, 30 s OFF, 5 cycles, high frequency). Protein concentration was measured using the Pierce BCA protein assay Kit (Thermo Fisher) according to the manufacturer’s protocol. Color change was measured at 564 nm using SpectraMax 190 plate reader (Molecular Devices) and the SpectraMax software (SoftMax Pro 7.1). Protein samples were prepared with 5X laemmli-buffer (300 mM Tris–HCl pH 6.8, 10% SDS, 50% glycerol, 5% β-Mercaptoethanol) and boiled at 95 °C for 10 min. Equal amounts of protein were used to run SDS–PAGE using the NuPAGE™ system (Thermo Fisher). All immunoblots were run on 4–12% Bis–Tris gels with NuPAGE™ MOPS SDS running buffer (20x). SDS–PAGE was performed according to manufacturer’s protocol. Gels containing separated proteins were blotted onto methanol pre-activated PVDF membranes (0.2 µm) at 10 V for 14 h at 4 °C in NuPAGE™ transfer puffer (20x) with 10% Methanol. Afterward, membranes were blocked for 1 h at RT with 5% milk in Tris-buffered saline supplemented with Tween (TBS-T; TBS supplemented with 0.1% Tween). For antibodies against phosphorylated proteins, membranes were blocked with 5% BSA in TBS-T. Subsequently, the membranes were incubated with primary antibodies diluted in TBS-T 5% milk/BSA overnight: anti-HA, anti-LAMP1, anti-LC3, anti-p62, anti-STAT1, anti-P-STAT1 and anti-β-actin (Online Resource Table 4). On the next day, the membranes were washed three times for 5 min with TBS-T, followed by incubation with secondary antibody (diluted in TBS-T 5% milk/BSA) for 1 h at RT (Online Resource Table 4). Blots were developed using ECL blotting solution (Amersham), and chemiluminescence was detected on an automated detection system (ChemiDoc MP, Biorad). To visualize total protein, membranes were incubated in Direct Blue (DB) 71 (Sigma-Aldrich) stain solution (0.008% DB71 in 40% ethanol, 10% acetic acid) for 5 min. Blot signals were quantified densitometrically using ImageJ (version 1.54d). The signal was normalized to the corresponding signal of β-actin.

### RNA isolation and quantitative real-time PCR (qPCR)

For gene expression analysis, RNA was isolated from iMGL at day 28 of differentiation. Cells were lysed in 0.5–1 ml QIAzol Lysis Reagent (Qiagen) and incubated for 5 min at RT. 100–200 μl chloroform (Carl Roth) was added and the suspension was mixed vigorously by shaking manually for 15 s. The suspension was incubated for 3 min at RT and centrifuged for 15 min at 12,000 g and 4 °C. The upper aqueous phase containing RNA was transferred into a new 1.5 ml tube and an equal volume of 70% ethanol was added. RNA extraction was continued using the RNeasy Mini Kit (Qiagen) following the manufacturer’s instructions. gDNA digestion by DNase I treatment was performed for bulk RNA sequencing. The purity and final concentration of the isolated RNA were measured using a Nanophotometer NP80™ (Implem). Isolated RNA was stored at  – 80 °C. For cDNA synthetization, QuantiTect Reverse Transcription Kit (Qiagen) was used according to manufacturer’s instructions. PCR reactions were set up with respective primers (Online Resource Table 5) using SYBR Green PCR Master Mix (Thermo Fisher) according to manufacturer’s instructions. For each PCR reaction, 2.5 ng of cDNA were used. Signal was analyzed on a Roche LightCycler 480 real-time PCR platform. Ct values were normalized to two housekeeping genes (GAPDH and HRPT) and expression values relative to untreated samples were calculated using the ddCt method [55].

### RNA sequencing

Isolated RNA from three SPG11 and four control iMGL, each untreated and IFNγ treated, underwent paired-end RNA sequencing with Poly-A selection using an Illumina NovaSeq, 2 × 150 bp configuration (Genewiz Germany). Samples were sequenced to a depth of 40,000,000 reads. Fastq files were first trimmed using Trimmomatic (v0.39) [10] and aligned to the human genome (GRCh38) using STAR (v2.7.9a) [26]. The feature Counts module within the Subread package (version 27) was utilized to assign reads to genes in the gencode annotation (version 26). In every sample, > 80% of reads are mapped uniquely to the human genome. Reads Per Kilobase of transcript, per Million mapped reads (RPKM) were calculated from the obtained counts to normalize for gene expression. Only genes with a mean RPKM value of 1 across the data set were considered for further analysis. PCA analysis was performed on RPKM values in Python 3 (v3.9.7). DESeq2 was used to determine differentially expressed genes (v1.34.0) [58]. DESeq2 output was filtered for adjusted *p* < 0.05 and |log2(fold change)|> 1. All downstream analyses were performed in Python 3 (v3.9.7) and graphs were generated using seaborn (v0.11.2).

### Statistical analysis

Statistical analysis was performed using GraphPad Prism 9 Software (GraphPad Software Inc.). Normal distribution was examined using the Shapiro–Wilk test. When data were normally distributed, a two-sided unpaired *t *test was used to compare two groups, and a one-way ANOVA test to compare more than two groups, followed by Bonferroni’s post-hoc test. For determining differences between data that were not normally distributed, Mann–Whitney *U* test was conducted. For grouped analyses (e.g., NT vs. IFNγ in Ctrl vs. SPG11), two-way ANOVA was performed. *P* values < 0.05 were considered significant (*P < 0.05, ** *P* < 0.01, *** *P* < 0.001, **** *P* < 0.0001).

## Results

### SPG11 human *postmortem* brain: neurodegeneration and accumulation of immune cells

**Fig. 1:**
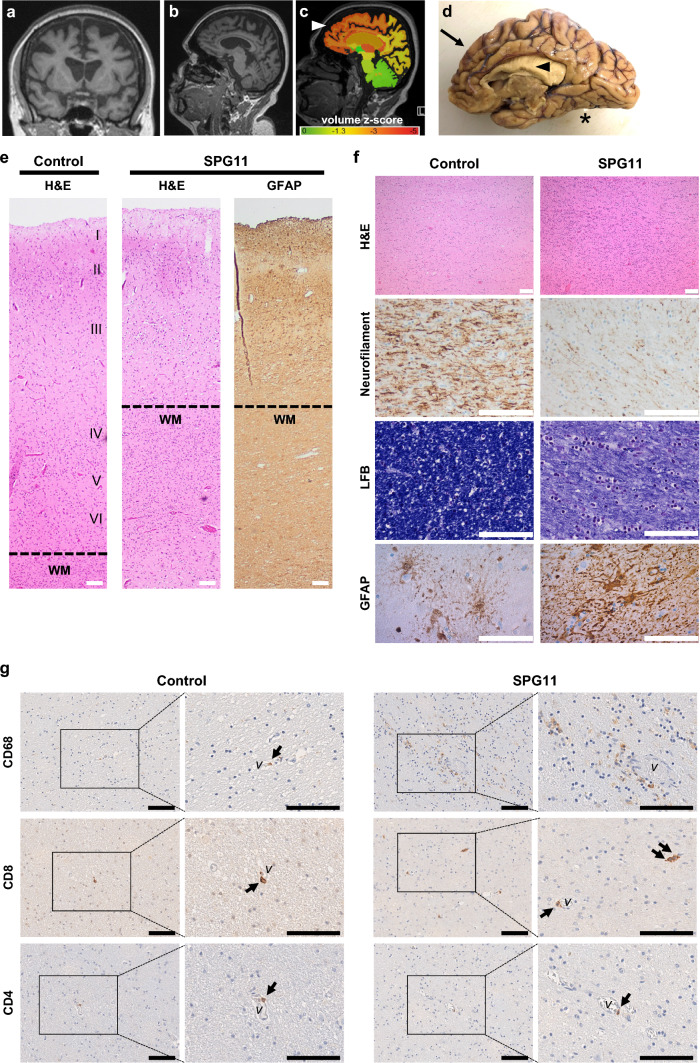
Neurodegeneration and immune cell activation in SPG11 *postmortem* brain tissue. **a–c** MRI of SPG11 (UKER) patient acquired 5 years before death, demonstrating a severe frontoparietal and callosal atrophy on **a** coronal, **b** parasagittal and **c** morphometric analysis, with red indicating lower brain volume (arrowhead). **d** Macroscopic medial view of the *postmortem* brain of the UKER patient with an arrow indicating frontal and parietal atrophy and arrowhead indicating thinning of the corpus callosum. *cerebellum was removed. **e** Neuropathological characterization showing profound atrophy of the parietal cortex on H&E predominantly involving layers III–VI along with reactive astrogliosis (GFAP) compared to a representative control tissue derived from an age- and sex-matched individual deceased without neurological disorder. Scale bar: 100 µm. **f** Internal capsule of SPG11 brain displaying increased cell numbers (H&E) and loss of neurofilament protein and myelin (LFB), as compared to the matched control. Astrogliosis (GFAP) is present within the putamen. Scale bar 100 µm. **g** Increase in CD68^+^ myeloid cells and CD8^+^ cells in SPG11, whereas CD4^+^ cells were exclusively observed in proximity to the vasculature (*v*) in the capsula interna of the SPG11 tissue. Scale bar 100 µm. *WM* white matter; *LFB* luxol fast blue

A very limited number of neuropathological analyses of five SPG11–HSP patients have been reported [25, 38, 62]. We, therefore, analyzed a previously unpublished *postmortem* brain from a late-stage SPG11 patient from the Erlangen movement disorder clinic (UKER), whose clinical history was reported earlier [39]. Briefly, this Caucasian female had a complex HSP, with a disease onset at age 31, progressive spasticity of the lower extremities and distal amyotrophy, and death 20 years later due to dysphagia and asphyxia. Biallelic pathogenic variants in the *SPG11* gene had been detected by molecular genetic testing (see methods and Online Resource Table 3) [39]. Cerebral MRI had shown severe frontoparietal and corpus callosum atrophy (Fig. 1a–c). Macroscopical evaluation of the *postmortem* brain confirmed this frontoparietal atrophy (Fig. 1d). Microscopically, the *postmortem* brain was characterized by severe neuronal loss predominantly in the frontal cortex and moderate neuronal loss in the parietal cortex, especially in cortical layers IV and V, while temporal and occipital cortex were only mildly affected. This was accompanied by astrogliosis and neuritic loss in white matter areas (Fig. 1e). In the capsula interna, particularly, neurofilament protein expression was severely reduced in addition to neuronal loss (Fig. 1f). Moreover, there was a marked reduction of myelin and a profound level of reactive gliosis (Fig. 1f). Additional observations included mild loss of neurons in the cerebellum and pons, but severe loss of neurons at all levels of the spinal cord, spanning the complete cross-sectional area. We next addressed the presence of infiltrated or reactive immune cells and detected an accumulation of CD68^+^ myeloid cells and CD8^+^ T cells. In contrast, there were no marked alterations in the frequency of CD4^+^ cells, and they were exclusively localized in the perivascular space (Fig. 1g). In summary, the presented SPG11 *postmortem* case shows classical signs of SPG11–HSP neurodegeneration, and, in addition, an accumulation of immune cells.

### Microgliosis and characteristics of disease-associated microglia (DAM) in the SPG11 *postmortem* brain

**Fig. 2:**
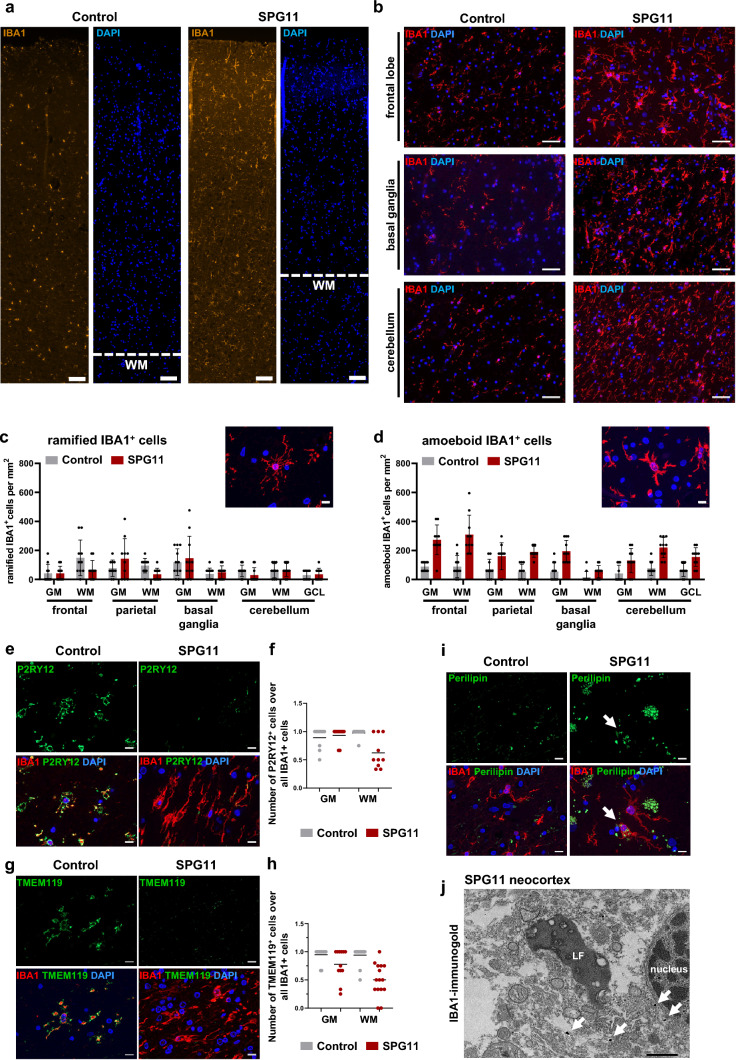
Activation of myeloid cells and cell-intrinsic SPG11 pathology. **a** Representative immunofluorescence for IBA1 and DAPI of the cortex of one control without neurological disease and the SPG11 (UKER) patient. Scale bar 100 µm. **b** Representative immunofluorescence for IBA1 and DAPI in different *postmortem* brain regions. Scale bar 50 µm. **c + d** Density of IBA1^+^ cells with ramified **c** or amoeboid **d** morphology comparing control and SPG11 in different brain regions, manually quantified per mm^2^ from two sections per condition according to depicted example images. Each dot represents one randomly selected field of view, analyzed blinded for genotype. Data presented as mean ± SD. Scale bars 10 µm. **e** Representative immunofluorescence images of the frontal lobe white matter (WM), stained for P2RY12 and IBA1 demonstrating fewer P2RY12^+^ cells in SPG11 compared to the control. Scale bar 10 µm. **f** Ratio of P2RY12^+^ over all IBA1^+^ myeloid cells in WM and grey matter (GM). Each dot represents cell counts from one field of view (0.014 mm^2^). Data presented as mean. *n* = 10. **g** Representative immunofluorescence images of frontal lobe WM, stained for TMEM119 and IBA1 demonstrating fewer TMEM119^+^ cells in SPG11 compared to control. Scale bar 10 µm. **h** Ratio of TMEM119^+^ over all IBA1^+^ myeloid cells in GM and WM. Each dot represents cell counts from one field of view (0.014 mm^2^). Data presented as mean. *n* = 10. **i** Representative immunofluorescence for IBA1 and perilipin in control and SPG11 cortex, demonstrating perilipin accumulation within an SPG11 IBA1^+^ myeloid cell (arrow). Scale bar 10 µm. **j** Ultrastructural evaluation of SPG11 frontal cortex showing abundant lipofuscin (LF) granules within a myeloid cell labeled with IBA1-immunogold (arrows). Scale bar 2 µm. *GM* grey matter; *WM* white matter; *GCL* granular cell layer;﻿ *LF* lipofuscin

We next performed an in-depth analysis of myeloid cells in the SPG11 case described above. IBA1^+^ cells were abundant in the parietal cortex and all other analyzed brain regions (Fig. 2a, b). Based on their morphology, IBA1^+^ cells were further classified into ramified homeostatic-like and amoeboid reactive-like (Fig. 2c, d) and compared to an age- and sex-matched control deceased from pulmonary embolism without neurological disease. While the number of ramified homeostatic-like IBA1^+^ cells were unchanged in SPG11 brain (Fig. 2c), there was a marked increase of amoeboid reactive-like IBA1^+^ cells in all analyzed SPG11 brain regions (Fig. 2d).

To substantiate these findings, we analyzed myeloid cells in two additional *postmortem* cases of SPG11–HSP that had been banked in Belgium (case BG2, previously reported in Denora et al. 2016 [25]) and at the University of Washington (case UWA, precise clinical description will be reported elsewhere; Scherpelz et al., in review). Comparing CD8^+^ T cells to a matched control without neurological diseases included by the respective center, there was an apparent increase in infiltrating CD8^+^ T cells in the BG2 case (Online Resource Fig. 1a). Increased numbers of CD68^+^ myeloid cells and accumulation of amoeboid IBA1^+^ myeloid cells were also present in both, the BG2 and UWA SPG11 *postmortem* brain tissues (Online Resource Fig. 1a, b), confirming our findings in the UKER case, and prompting a more detailed analysis of IBA1^+^ myeloid cells in the well-preserved tissue of the UKER case.

P2RY12 and TMEM119 are markers of homeostatic microglia and are downregulated in disease-associated microglia (DAM) [23]. Compared to control brain tissue, numbers of IBA1^+^P2RY12^+^ double positive cells were predominantly decreased in the frontal and parietal lobe white matter regions of the SPG11 *postmortem* tissue (Fig. 2e, f; Online Resource Fig. 2a). This decrease was not observed in SPG11 basal ganglia and cerebellum, where P2RY12 expression levels were comparable to control tissue (Online Resource Fig. 2a). Several brain regions in the SPG11 patient had decreased numbers of IBA1^+^TMEM119^+^ double positive cells, compared to the control brain, especially within the white matter of the frontal and parietal lobes (Fig. 2g, h; Online Resource Fig. 2b). Since SPG11 pathology has been related to failure of the autophagic–lysosomal machinery and of lipid clearance [15, 82, 99], we next analyzed the expression of perilipin, a surface marker protein of lipid droplets. Perilipin-loaden cells were frequently observed in the SPG11 brain, but not in the control brain. Specifically, we detected IBA1^+^Perilipin^+^ cells, indicating lipid accumulation within SPG11 myeloid cells (Fig. 2i; Online Resource Fig. 2c). Matching previous reports of SPG11 neuropathology [25, 38, 62], there were abundant amounts of lipofuscin. Using immunogold labeling for IBA1, we observed that these lipofuscin accumulations were also present within SPG11 myeloid cells (Fig. 2j). IBA1^+^ cells of both ramified and amoeboid morphology did not cluster to the vasculature but were distributed throughout the parenchyma (Online Resource Fig. 2d).

Altogether, neuropathology in the SPG11 (UKER) case exhibited astrogliosis, infiltration of CD8^+^ T cells, and widespread severe microgliosis which was also detected in two additional SPG11 *postmortem* brains. SPG11 IBA1^+^ myeloid cells were characterized by downregulation of homeostatic markers and accumulation of perilipin.

### Peripheral inflammation in SPG11 patients

**Fig. 3:**
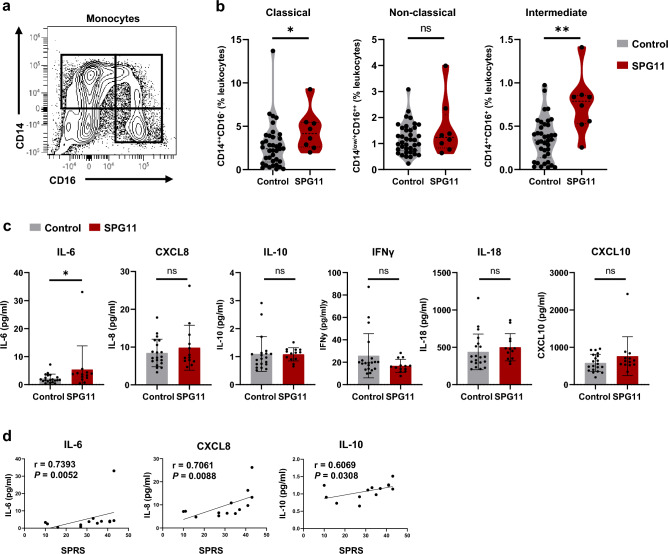
Evaluation of peripheral inflammation in additional SPG11–HSP patients. **a** Flow cytometric (FC) gating strategy to divide monocytes into classical CD14^++^CD16^−^ (upper left), non-classical CD14^low/+^CD16^++^ (bottom right) and intermediate CD14^++^CD16^+^ (upper right) subpopulations. **b** Quantification of classical (CD14^++^CD16^−^), non-classical (CD14^low/+^CD16^++^) and intermediate (CD14^++^CD16^+^) monocytes in the peripheral blood as a percentage of total leukocytes, obtained by FC analysis of PBMCs derived from SPG11 patients and controls. *n*(SPG11) = 8, *n*(control) = 38. ns *P* > 0.05, *P < 0.05, **P < 0.01, according to a non-parametric Mann–Whitney *U* test. **c** Cytokine concentrations in the serum of controls (grey) and SPG11–HSP patients (red) measured by MSD multiplex immunoassay. ns *P* > 0.05, * *P* < 0.05, according to a non-parametric Mann–Whitney *U* test. Data presented as mean ± SD.* n*(SPG11) = 13, *n*(control) = 20. **d** Correlation of serum cytokine concentrations in SPG11–HSP patients with clinical SPRS values. Correlation coefficients r and *P* value according to a Spearman correlation. *FC* Flow cytometry, *SPRS* spastic paraplegia rating scale, *ns* not significant

Since the peripheral immune response and its crosstalk with neuroinflammation is of increasing relevance in other neurodegenerative diseases, we additionally investigated peripheral immunity in a larger SPG11 cohort. Specifically, we studied monocyte subpopulations and peripheral blood levels of inflammatory cytokines, as potential modulators of CNS immune cells, comparing SPG11–HSP patients to age- and sex-matched controls (Online Resource Tables 1 and 2). FC analysis of monocyte subsets revealed increased levels of classical (CD14^++^CD16^−^) and intermediate (CD14^++^CD16^+^) monocytes in SPG11–HSP patients (*n* = 8) compared to controls (*n* = 38), while the percentage of non-classical (CD14^low/+^CD16^++^) monocytes was unchanged (Fig. 3a, b; Online Resource Fig. 3). Measurements of serum from SPG11–HSP patients (*n* = 13) compared to matched controls (*n* = 20) showed increased levels of the proinflammatory cytokine IL-6, while levels of additional cytokines were unchanged (Fig. 3c). Interestingly, within the SPG11–HSP cohort, there was a positive correlation of IL-6 levels to disease severity as measured by the SPRS (Spastic Paraplegia Rating Scale; Fig. 3d). Disease severity of SPG11 patients also correlated with CXCL8 and IL-10 serum concentrations (Fig. 3d).

In conclusion, the observation of an increase in classical and intermediate monocytes and elevated levels of proinflammatory cytokines correlating with disease severity in larger cohorts of patients strongly indicate proinflammatory changes in SPG11–HSP.

### Generation of SPG11–HSP patient-specific induced microglia-like cells

**Fig. 4:**
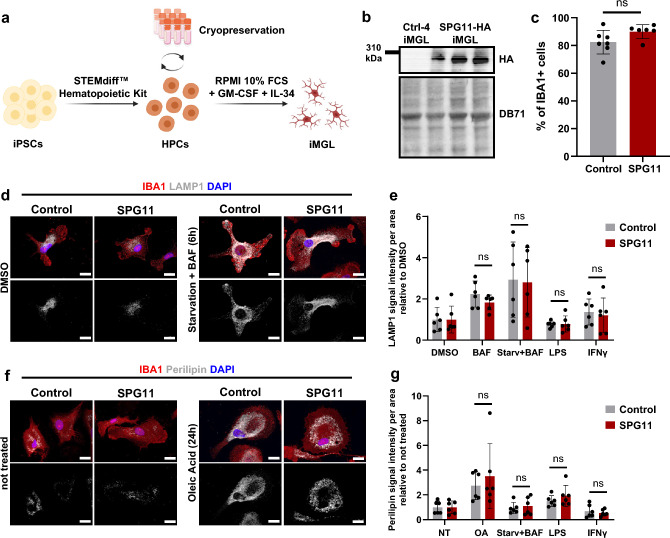
SPG11 iPSCs efficiently differentiate into induced microglia-like cells (iMGL) without excessive lysosome or lipid accumulation. **a** Schematic representation of iPSC differentiation into iMGL via the HPC state. HPCs were generated using STEMdiff™ Hematopoietic Kit and cryopreserved. Further differentiation into iMGL was performed for 2 weeks in RPMI containing 10% FCS, 10 ng/mL GM–CSF and 50 ng/ml IL-34. **b** Immunoblotting of a bi-allelic HA-tagged *SPG11* iPSC line that was differentiated into iMGL (SPG11-HA iMGL) using an antibody against HA. Three biological replicates of SPG11–HA are depicted. The HA-tag was detected at the size of spatacsin (~ 280 kDa) and was absent in the parental non-tagged iMGL control line (Ctrl-4). Total protein was stained by DB71 as a loading control. **c** Percentage of IBA1^+^ cells determined by FC analysis. Each dot represents one cell line. Data are presented as mean ± SD. *n*(SPG11) = 6, *n*(control) = 7. *ns P* > 0.05, according to a non-parametric Mann–Whitney *U* test. **d** Representative immunofluorescence of control and SPG11 iMGL for IBA1 and LAMP1. Left: vehicle-treated control and SPG11 iMGL. Right: iMGL were starved by serum depletion for 24 h in addition to BafilomycinA1 treatment (100 nM for 6 h). Scale bar: 10 µm. **e** LAMP1 fluorescence signal intensity, normalized to DMSO control. LPS: 100 ng/µl for 24 h. IFNγ: 10 ng/µl for 24 h. Each dot represents the mean normalized signal intensity per cell line (derived from ten images per line); bars represent mean ± SD. *n* = 6. *ns P* > 0.05, according to a two-way ANOVA with Bonferroni’s multiple comparison test. **f** Representative immunofluorescence of control and SPG11 iMGL for IBA1 and perilipin, both unstimulated and after exposure with Oleic acid (200 µM for 24 h). Scale bar: 10 µm. **g** Perilipin fluorescence signal intensity, normalized to untreated controls. Control iMGL are depicted in grey and SPG11 iMGL in red. Each dot represents the mean perilipin signal intensity per cell line (derived from ten images per line); bars represent mean ± SD. *n* = 6. *ns P* > 0.05, according to a two-way ANOVA with Bonferroni’s multiple comparison test. *iPSC* induced pluripotent stem cells; *iMGL* induced microglia-like cells; *HPC* hematopoietic stem cells; *FCS* fetal calf serum; *FC* Flow cytometry; *ns* not significant; *BAF* BafilomycinA1; *LPS* Lipopolysaccharide; *OA* Oleic Acid; *Starv* Starvation; *NT* non-treated

To test for cell-intrinsic alterations of microglia upon loss of *SPG11*-encoded spatacsin function, induced microglia-like cells (iMGL) were differentiated from six patient- and six control-derived iPSC lines (Fig. 4a) [52]. Details of the iPSC lines are listed in Online Resource Table 3. We first validated the expression of spatacsin within iMGL. Due to the limited reliability of available spatacsin antibodies, we used a bi-allelically endogenously tagged *SPG11*–human iPSC line (SPG11–HA) that had been previously generated via clustered regularly interspaced short palindromic repeats (CRISPR)/Cas9-mediated knock-in of an HA tag [51]. After differentiating SPG11–HA iPSC together with the parental iPSC line (Ctrl-4) into iMGL, HA-tagged spatacsin showed a strong signal at the expected size in SPG11–HA iMGL (Fig. 4b).

Both from control and SPG11 iPSC, iMGL were successfully generated. FC analysis for IBA1 showed differentiation ratios of approximately 70–90%, without differences between control and SPG11 iMGL lines (Fig. 4c and Online Resource Fig. 4a). Both control and SPG11 iMGL expressed IL-1β upon LPS treatment (Online Resource Fig. 4b).

Loss of spatacsin function has been closely linked to accumulation of lysosomes and lipids in mouse models, primary patient fibroblasts and iPSC-derived motor neurons [14, 34, 82, 98, 99]. We, therefore, first compared the lysosomal compartment between control and SPG11 iMGL, both under basal conditions and when applying different stimulation paradigms. To this end, we quantified lysosomal compartments by analyzing LAMP1^+^ area of iMGL under basal conditions, upon BafilomycinA1 treatment, upon starvation combined with BafilomycinA1, upon stimulation with LPS, and upon stimulation with IFNγ (Fig. 4d, e; Online Resource Fig. 4c, d). As expected, LAMP1 signal intensity increased upon dual serum deprivation and BafilomycinA1 treatment, but there were no differences between control and SPG11 iMGL (Fig. 4d, e). Accordingly, the expression of LAMP1 as well as p62, as evaluated by Western blot, was unchanged in SPG11 iMGL and controls (Online Resource Fig. 4g, h).

Based on the neuropathology finding of perilipin accumulation in SPG11 (Fig. 2i), control and SPG11 iMGL were also stained for the lipid marker perilipin. Under baseline conditions, perilipin^+^ lipid droplets were similarly distributed in SPG11 iMGL and control, and there were no quantitative differences in signal intensity per cell (Fig. 4f, g; Online Resource Fig. 4f). As a positive control, incubation with oleic acid led to increased perilipin levels, both in SPG11 and control. Similarly, there was no difference upon inhibition of lysosomal degradation induced by serum depletion and BafilomycinA1 treatment (Fig. 4f, g). Neither stimulation of iMGL with LPS nor with IFNγ led to alterations of perilipin expression (Fig. 4g; Online Resource Fig. 4e). In conclusion, we did not detect lysosomal abnormalities or increased lipid accumulations in SPG11 iMGL.

### IFNγ triggers STAT1-dependent hyperactivation of SPG11 iMGL

**Fig. 5:**
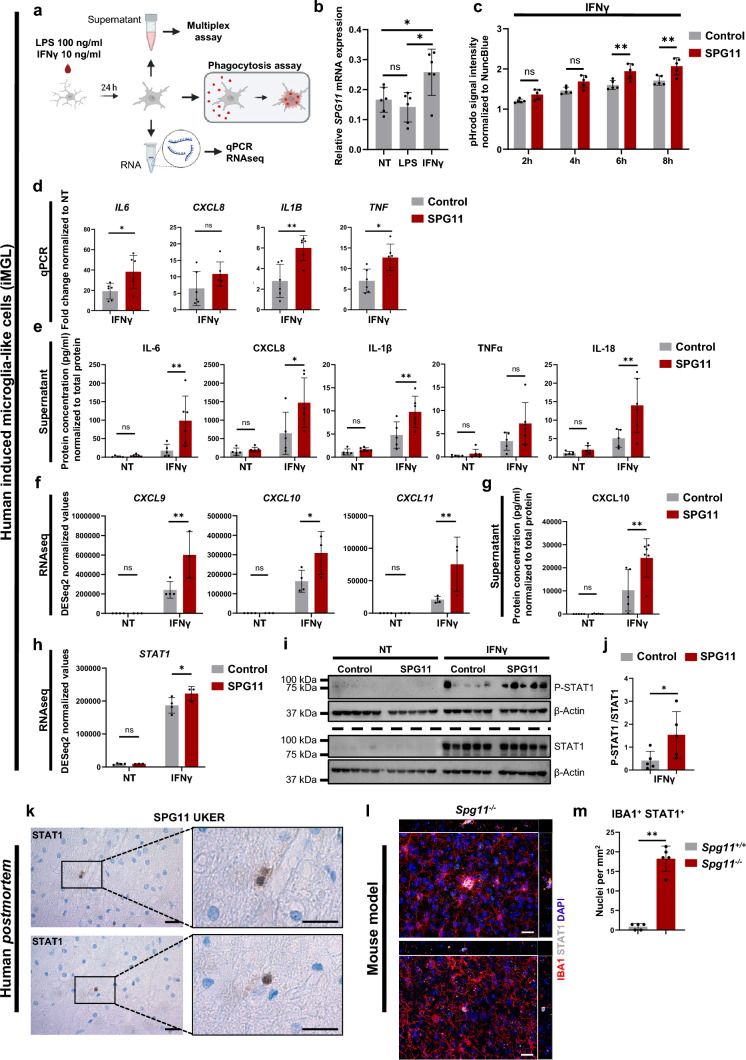
Increased STAT1-dependent IFNγ signaling in SPG11 microglia results in excessive upregulation of proinflammatory cytokines and chemokines. **a** Schematic representation of the experimental paradigm. iMGL were treated with LPS (100 ng/ml) or IFNγ (10 ng/ml) for 24 h before supernatants, RNA expression or phagocytosis were analyzed. **b** In control iMGL, relative expression of *SPG11* mRNA is upregulated upon IFNγ but not LPS stimulation. *n* = 6. Data are presented as mean ± SD. *P* value according to one-way ANOVA with Bonferroni’s multiple comparison test. **c** Signal intensity of bacterial pHrodo particles in IFNγ treated control (grey) and SPG11 (red) iMGL, normalized to cell density measured by NuncBlue. Fluorescence signal was measured at 2 h, 4 h, 6 h and 8 h after incubation with bacterial particles.* n* = 5. *P* value according to a two-way ANOVA with Bonferroni’s multiple comparison test. **d** Relative gene expression of IFNγ treated control (grey) and SPG11 (red) iMGL normalized to the expression of the untreated condition for each line. *n* = 6. Each data point represents the mean derived from two independent technical replicates. Each bar indicates the mean ± SD for each condition. *P* value according to a non-parametric Mann–Whitney *U* test. **e + g** Cytokine concentrations in the supernatant of untreated vs. IFNγ treated control and SPG11 iMGL normalized to total protein.* n*(control) = 5;* n*(SPG11) = 6. Data are presented as mean ± SD. *P* value according to a two-way ANOVA with Bonferroni’s multiple comparison test. **f + h** DESeq2 normalized gene expression values of untreated and IFNγ treated iMGL (control in grey and SPG11 in red). *n*(control) = 4; *n*(SPG11) = 3. Data presented as mean ± SD.* P* value according to a two-way ANOVA with Bonferroni’s multiple comparison test. **i** Immunoblotting of control and SPG11 iMGL, untreated or treated with IFNγ: 10 ng/µl for 24 h, using antibodies against phosphorylated STAT1 (P-STAT1) and total STAT1. β-actin was used as a loading control. Different membranes are separated by a dashed line. Samples on both membranes were derived from the same experiment and gels as well as blots were processed in parallel. **j** Relative P-STAT1 expression normalized to total STAT1 in control (grey) and SPG11 (red) iMGL upon IFNγ stimulation. P-STAT1 and STAT1 signals were first normalized to respective β-actin signals. *n* = 5. Data presented as mean ± SD. *P* value according to a non-parametric Mann–Whitney *U* test. **k** Two representative STAT1 DAB stainings of the SPG11 UKER *postmortem* parietal lobe. Scale bar 20 µm. **l** Two representative microscopic z-stack images of *Spg11*^*−/−*^ frontal lobe stained for IBA1, STAT1 and DAPI. Scale bar: 20 µm. **m** Quantification of IBA1^+^ cells that show a nuclear STAT1 signal. *n*(*Spg11*^+*/*+^) = 5; *n*(*Spg11*^*−/−*^) = 5. Each dot represents the mean of cells within five random fields of view (0.15 mm^2^). Data presented as mean ± SD. *P* value according to a non-parametric Mann–Whitney *U* test. *ns* P > 0.05; * P < 0.05; ** P < 0.01; *NT* non-treated; *STAT1* Signal Transducer and Activator of Transcription 1

Given the expression of spatacsin in iPSC-derived iMGL (Fig. 4b) and the neuropathological observation of lipofuscin accumulation in SPG11 myeloid cells (Fig. 2j), we next studied the inflammatory response of SPG11 iMGL. To this end, we applied LPS (100 ng/ml) or IFNγ (10 ng/ml) for 24 h (Fig. 5a). Interestingly, *SPG11* mRNA expression in control iMGL was significantly increased upon IFNγ stimulation, whereas LPS stimulation did not alter *SPG11* expression (Fig. 5b), indicating a role of *SPG11* in IFNγ-mediated activation of iMGL. To determine whether SPG11 iMGL respond differently to IFNγ, we first quantified their phagocytic capacity, as one of the main functions of microglia. While untreated SPG11 iMGL and controls had comparable levels of phagocytosed fluorescent bacterial particles, increased phagocytosis activity was present in IFNγ treated SPG11 iMGL compared to controls, particularly after 6 h and 8 h (Fig. 5c; Online Resource Fig. 5a, b). As a negative control, both control and SPG11 iMGL phagocytosed fewer bacterial particles upon Cytochalasin D treatment, which is known to block phagocytosis (Online Resource Fig. 5c).

In the next step, we examined gene and protein expression of proinflammatory cytokines, such as IL-6, which were increased in the serum of SPG11 patients (Fig. 3c). Both under basal conditions and upon LPS stimulation, control and SPG11 iMGL exhibited no difference in inflammatory gene expression (Online Resource Fig. 5d, e). IFNγ stimulation, however, induced significant upregulation of *IL6*, *IL1B* and *TNF* in SPG11 iMGL compared to controls (Fig. 5d). Moreover, significantly increased levels of IL-6 and IL-1β, as well as CXCL8 and IL-18 were present in the supernatant of IFNγ treated SPG11 iMGL (Fig. 5e), while cytokine concentrations after LPS stimulation were unchanged (Online Resource Fig. 5f). To identify additional dysregulation of genes and pathways, we next performed RNAseq of control vs. SPG11 iMGL. Principal component analysis (PCA) showed a clear clustering of untreated vs. IFNγ treated iMGL, but no overall difference between control and SPG11 iMGL (Online Resource Fig. 5g). The number of differentially expressed genes in control vs. SPG11 was within a similar range in the untreated and IFNγ treated conditions (Online Resource Fig. 5h). When focusing on specific genes of interest, we validated the increased upregulation of *IL6* in IFNγ treated SPG11 iMGL as well as increased *SPG11* expression upon IFNγ (Online Resource Fig. 5i), matching the qPCR results (Fig. 5b, d). *SPG11* upregulation was also present in SPG11 patient-derived iMGL although at lower absolute reads, compatible with their loss of function variants in *SPG11* (Online Resource Fig. 5i). Furthermore, IFNγ exposure induced higher upregulation of *CXCL9*, *CXCL10* and *CXCL11* in SPG11 iMGL compared to controls (Fig. 5f). The differential expression of these closely related chemokines was also confirmed by qPCR (Online Resource Fig. 5j). Compared to controls, significantly increased CXCL10 concentrations were observed in the supernatant of IFNγ treated SPG11 iMGL (Fig. 5g).

To further obtain insights into underlying mechanisms, we next addressed which step within the IFNγ signaling cascade is altered in SPG11 iMGL. Activation of Signal Transducer and Activator of Transcription 1 (STAT1) is one major downstream effect of IFNγ. Indeed, *STAT1* gene expression was significantly increased in IFNγ treated SPG11 iMGL as compared to controls (Fig. 5h). To further assess STAT1 signaling activity, IFNγ dependent phosphorylation of STAT1 was evaluated by Western blot. In both control and SPG11 iMGLs, upregulation of STAT1 and phosphorylated STAT1 was detected after IFNγ stimulation, compared to very low levels in the untreated conditions (Fig. 5i). Remarkably, levels of phosphorylated STAT1 were significantly higher in SPG11 than in control iMGLs, indicating an increased induction of STAT1 signaling activity (Fig. 5j). We next aimed to analyze STAT1 in vivo. Correlating to the in vitro findings, STAT1^+^ cells were observed in the parietal lobe of the SPG11 UKER *postmortem* case while completely absent in the control sample (Fig. 5k, Online Resource Fig. 6). For an additional in vivo validation of increased IFNγ/ STAT1 signaling in SPG11, we analyzed an *Spg11*^–/–^ mouse model. We harvested brain tissue from 16-month-old *Spg11*^+/+^ and *Spg11*^–/–^ mice that are characterized by an HSP-like phenotype with loss of cortical neurons and accumulation of autofluorescent material [46, 99]. There was a profound microgliosis in the frontal lobe of *Spg11*^–/–^, and we also detected accumulation of autofluorescent material within IBA1^+^ microglia, comparable to the SPG11 *postmortem* findings (Online Resource Fig. 6b–e). The number of IBA1^+^ microglia presenting STAT1^+^ nuclei was significantly increased in *Spg11*^*−/−*^ by 17-fold, as only very few STAT1^+^ microglia were detected in *Spg11*^+/+^ (Fig. 5l, m; Online Resource Fig. 6f).

Taken together, we demonstrate an interplay between IFNγ and SPG11, including upregulation of *SPG11* upon IFNγ stimulation and a hyperactivation phenotype of SPG11 iMGL characterized by increased phagocytosis and cytokine expression. Moreover, STAT1 signaling was identified as a potential key mechanism in SPG11 iMGL, but also in the human SPG11 *postmortem* case and in a murine in vivo model of SPG11–HSP.

### Ruxolitinib prevents hyperactivation of SPG11 iMGL and induced toxicity in SPG11 neurons

**Fig. 6:**
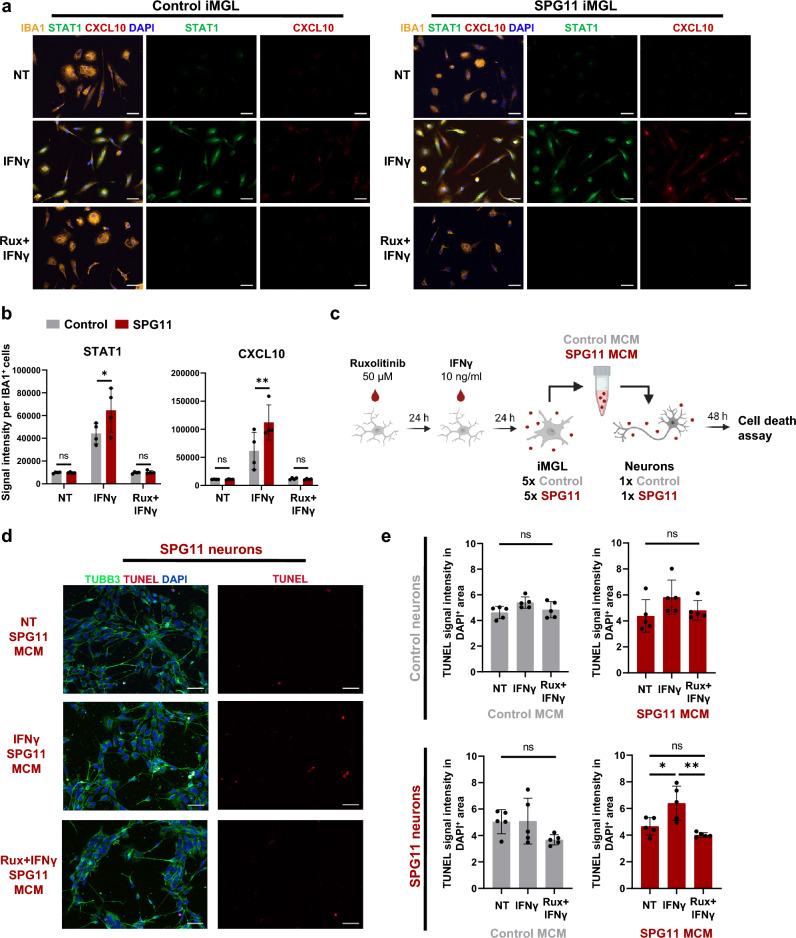
Ruxolitinib inhibits SPG11 iMGL hyperactivation and rescues toxicity induced in SPG11 neurons. **a** Representative immunofluorescence of control and SPG11 iMGL (IFNγ: 10 ng/µl for 24 h, Ruxolitinib: 50 µM for 24 h before IFNγ treatment) stained for IBA1, STAT1 and CXCL10. Scale bar 50 µm. **b** Quantification of mean fluorescence intensity of STAT1 and CXCL10 within IBA1^+^ control (*n* = 4) and SPG11 (*n* = 4) iMGL. Each dot represents the mean of cells within five random fields of view (0.15 mm^2^). Bars represent means ± SD. *P* value according to two-way ANOVA with Bonferroni’s multiple comparison test*.* **c** Schematic representation of the experimental paradigm. Control and SPG11 iMGL were either treated with Ruxolitinib and IFNγ, with IFNγ only or remained untreated. Microglia-conditioned media (MCM) was collected, diluted 1:1 with neuronal media and added to control and SPG11 neurons that were differentiated for 2 weeks from NPCs. After 48 h, cell death was measured by ICC. **d** Representative images of TUBB3 and DAPI staining in SPG11 neurons treated with MCM derived from non-treated (NT), IFNγ treated, and Ruxolitinib (Rux) and IFNγ treated SPG11 iMGL. Apoptotic cells were visualized by TUNEL assay. Scale bar: 50 µm. **e** Quantification of apoptotic cells by TUNEL signal intensity within the DAPI^+^ nuclei of TUBB3^+^ control (*n* = 1) and SPG11 (*n* = 1) neurons treated with MCM derived from control (*n* = 5) and SPG11 (*n* = 5) iMGL. Each dot represents the mean of cells within five random fields of view (0.15 mm^2^). Bars represent means ± SD. *P* value according to one-way ANOVA with Bonferroni’s multiple comparison test*. ns* *P* > 0.05; * *P* < 0.05; ** *P* < 0.01; *NT* non-treated; *Rux* Ruxolitinib; *MCM* microglia-conditioned media

As a next step, we evaluated ruxolitinib, an FDA- and EMA-approved Janus Kinase (JAK)-inhibitor, for its capacity to reduce IFNγ-induced hyperactivation of SPG11 iMGL. When testing different ruxolitinib concentrations in control iMGL prior to IFNγ stimulation, 50 and 100 µM showed the strongest capacity to block STAT1 relocalization to the nucleus and to prevent STAT1 and CXCL10 expression (Online Resource Fig. 7a, b). No increased cell death was observed upon ruxolitinib treatment (Online Resource Fig. 7c, d). In all used iMGL lines, control and SPG11, ruxolitinib pretreatment significantly reduced STAT1 and CXCL10 signal intensity after IFNγ stimulation (Fig. 6a, b; Online Resource Fig. 7e). Comparing SPG11 and controls, SPG11 iMGL displayed significantly higher STAT1 and CXCL10 signal intensity after IFNγ treatment, consistent with RNAseq and Western blot data (Fig. 5h–j).

Next, we assessed whether cytokines and chemokines secreted by SPG11 iMGL upon IFNγ stimulation have an effect on homologous and heterologous neurons. As depicted in Fig. 6c, control and SPG11 iPSC-derived neurons were treated with the supernatant from control and SPG11 iMGL (microglia-conditioned media [MCM]) that were either treated with Ruxolitinib and IFNγ, IFNγ only or remained untreated. Interestingly, supernatant derived from IFNγ treated SPG11 iMGL (SPG11–IFNγ–MCM) induced significantly increased cell death in SPG11 neurons compared to MCM from untreated SPG11 iMGL (NT–SPG11–MCM; Fig. 6d, e). The same trend was detectable in control neurons treated with SPG11–IFNγ–MCM, but without significance. Control-MCM did not lead to an increased neuronal cell death, neither in control nor in SPG11 neurons. Remarkably, this increase in cell death was prevented by ruxolitinib treatment prior to IFNγ stimulation (Fig. 6d, e). IFNγ alone did not increase cell death in control or SPG11 neurons. In contrast, treatment with sodium arsenite as a positive control significantly increased TUNEL signal intensity as a marker of cell death (Online Resource Fig. 8a, b).

In summary, hyperactivation of SPG11 iMGL was blocked by the approved JAK inhibitor ruxolitinib, which also effectively prevented increased cell death of neurons exposed to SPG11–IFNγ–MCM.

**Fig. 7:**
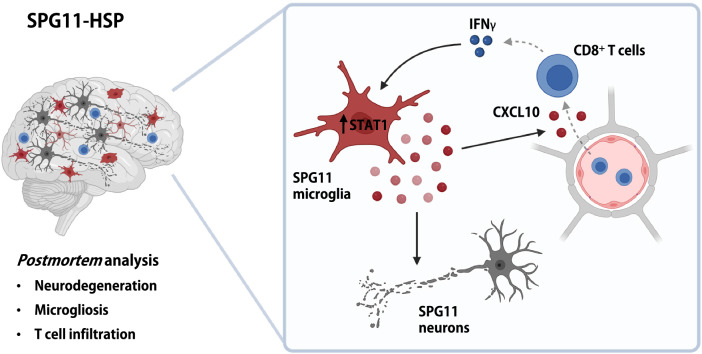
Paradigm of the proposed mechanism underlying neuroinflammation in SPG11–HSP. The *postmortem* analysis indicates that SPG11–HSP patients not only present neurodegenerative phenotypes but also neuroinflammatory characteristics, including microgliosis and infiltration of CD8^+^ T cells. Our in vitro data revealed that SPG11 iMGL are hyperreactive to IFNγ and produce STAT1-mediated increased levels of proinflammatory cytokines and chemokines that induced increased cell death in SPG11 neurons. In addition, SPG11 iMGL secrete more CXCL10, which is known to mediate infiltration of T cells into the CNS. As T cells are a major source of IFNγ in the brain, we hypothesize an IFNγ- and CXCL10-dependent, reinforcing vicious circle involving microglia and T-cell activation that may also contribute to neurodegeneration

.

## Discussion

Our findings delineate an important role of innate immunity in SPG11–HSP. The analysis of three SPG11 patients’ *postmortem* brains indicated an increase of IBA1^+^ myeloid cells with amoeboid reactive-like morphology. These cells also exhibited lipid accumulation and downregulation of homeostatic microglia markers. Moreover, peripheral inflammation was detected in a larger SPG11 cohort with increased levels of proinflammatory monocytes and cytokines. We show that IFNγ, but not LPS, induced hyperactivation in SPG11 iMGL, characterized by increased phagocytosis and upregulation of proinflammatory cytokines and chemokines. We additionally identified increased phosphorylation of STAT1 in SPG11 iMGL as a potential key mechanism underlying this hyperreactive phenotype. Increased STAT1 levels were also detectable in the SPG11 *postmortem* case and in *Spg11*^−/–^ mice. In addition, the JAK-inhibitor ruxolitinib rescued neurotoxicity induced by SPG11 iMGL conditioned media after IFNγ stimulation. Here, we describe a novel interplay between *SPG11* and IFNγ response, provide insights into cell-intrinsic and cell-extrinsic mechanisms, and introduce CNS immune cells as a potential therapeutic target in SPG11–HSP patients.

HSPs are a rare group of neurodegenerative disorders. For this reason, the availability of SPG11 *postmortem* brain tissue is very limited. So far, five *postmortem* cases have been reported [25, 38, 62]. All of these studies focused on neurodegenerative alterations that are in line with our observations. While general gliosis or astrogliosis was noted, no analysis has been presented on microglial alterations or infiltration of leukocytes. Here, we demonstrate for the first time an increase in immune cells in the brain of SPG11 patients, including infiltration of CD8^+^ T cells, accumulation of CD68^+^ cells and increased numbers of IBA1^+^ myeloid cells with amoeboid reactive-like morphology. These observations expand data from a recent report on an *Spg11*^−/−^ mouse model [41], which exhibited an age-dependent accumulation of CD8^+^ T cells in several brain regions along with increased numbers of CD11b^+^ microglia in *Spg11*^−/−^ mice at early symptomatic stages. The observed loss of myelinated fibers in the SPG11 (UKER) case in conjunction with an increase in CD68^+^ cells additionally indicates increased phagocytic activity of myeloid cells. Interestingly, loss of myelinated fibers of varying severity had been noted in all previous human SPG11 neuropathology reports [25, 38, 62].

Besides their amoeboid reactive-like morphology, IBA1^+^ myeloid cells in the SPG11 (UKER) *postmortem* case additionally presented a signature that has been previously referred to as DAM. This microglia subtype was first described in AD and is characterized by downregulation of homeostatic markers, such as CX3CR1, P2RY12 and TMEM119 [23, 45]. In general, DAM occur upon accumulation of neuronal apoptotic bodies and myelin debris, such as in ALS, AD, aging and demyelination [45, 50, 74, 101]. The precise function of the DAM population on neurodegeneration has not been elucidated, and both protective and toxic functions have been proposed [23]. In the presented SPG11 (UKER) case, DAM markers were predominantly detected in cerebral white matter, which is known to exhibit degenerative changes on MR imaging and previous *postmortem* studies [25, 28, 31, 90, 96, 102]. We further identified severe accumulation of lipid droplets and lipofuscin in *postmortem* IBA1^+^ myeloid cells, which is in line with the known dysregulation of lipid clearance and lysosomal function in SPG11. This lipid accumulation may result from phagocytosed material, but our iMGL findings suggest that intrinsic microglial dysfunction may also play a role. Increased accumulation of lipofuscin has been associated with age-related decline of neuronal and microglial function, particularly in AD and PD [30, 40, 59, 85, 87, 100].

Peripheral inflammation has emerged as a modulator of disease progression and neuropathology in several neurodegenerative diseases, such as AD, PD and other neurodegenerative disorders, where neuroinflammatory alterations are well-established [7, 36, 93]. In a cohort of SPG11–HSP patients, we here show increased levels of classical (CD14^++^CD16^−^) and intermediate (CD14^++^CD16^+^) monocytes. Classical monocytes have been reported to display a more proinflammatory phenotype producing the highest levels of various cytokines including IL-6 and being significantly more abundant in patients with multiple sclerosis (MS), PD and ALS [29, 33, 44, 103, 108]. The intermediate subpopulation is also reported to be increased in inflammatory conditions as well as in neurodegenerative diseases such as AD and indicates activation of peripheral innate immunity [32, 64, 83]. These monocyte subpopulations act primarily in a proinflammatory manner by expression of the highest levels of antigen presentation-related molecules and by secretion of TNFα, IL-1β, IL-6 and other cytokines [6, 53, 63, 103]. In the present study, we additionally observed a significant elevation of IL-6 that together with CXCL8 and IL-10 positively correlated with disease severity of SPG11 patients. Increased peripheral serum levels of proinflammatory cytokines such as IL-6, CXCL8, and IL-10 were reported not only in AD but also in PD and ALS patients [16, 42, 47, 79]. Although increased microglial activation in the cortex of HD patients has been associated with increased plasma concentrations of IL-1β, IL-6, CXCL8, and TNFα [75], the exact origin of these cytokines remains unclear and may derive from dysfunctional peripheral monocytes. The observed increase in peripheral inflammation in the present SPG11–HSP patient cohort is comparable to other neurodegenerative diseases, where the contribution of inflammation is well-established, supporting our findings of neuroinflammatory disease signatures in the limited *postmortem* tissue.

To determine cell intrinsic dysfunction of myeloid cells lacking functional *SPG11*, patient-derived iPSCs were converted into iMGL as previously established [52]. SPG11 iMGL did not show abnormal accumulation of lysosomes or lipids. This may be due to the lower vulnerability of phagocytic cells to lysosomal dysfunction and due to the early developmental phenotype of these cells [27]. Moreover, actual brain substrates may be necessary to recapitulate disease-associated phenotypes in iMGL, as shown recently for AD [27]. We identified IFNγ as a positive regulator of *SPG11* expression in iMGL, whereas the effects of LPS were not different between SPG11 and control lines. Interestingly, IFNγ was previously shown to induce LRRK2 expression in iPSC-derived neurons and microglia, proposing synergistic LRRK2/ IFNγ activation as a potential direct link between inflammation and neurodegeneration in LRRK2-related PD [68]. LRRK2–PD microglia as well as microglia from other neurodegenerative diseases additionally displayed increased phagocytic activity, paralleling our finding in IFNγ treated SPG11 iMGL [2, 67, 68]. Furthermore, increased expression of proinflammatory cytokines such as IL-6, IL-1β, and TNFα that we detected in IFNγ treated SPG11 iMGL, was also described for iPSC-derived microglia from patients with other neurodegenerative diseases, including PD and ALS [2, 4, 57, 68].

Receptor binding of IFNγ activates the canonical Janus kinase (JAK) and STAT1 pathways, which directly induces transcription of interferon-stimulated genes (ISGs) in macrophages [43]. Consistent with the excessive expression of cytokines and chemokines, IFNγ-induced STAT1 signaling activity was significantly increased in SPG11 iMGL. Moreover, in the SPG11 *postmortem* case as well as in *Spg11*^–/–^ mice, the number of IBA1^+^ microglia with STAT1^+^ nuclei was increased, indicating excessive IFNγ/ STAT1 signaling in SPG11 patients. Increased microglial STAT1 was additionally observed in a murine in vivo model of SPG11–HSP that shows an HSP-like phenotype. STAT1 is a major mediator of proinflammatory microglia activation, and inhibition of STAT1 signaling prevents microglia-dependent neurodegeneration [11, 17, 106]. In a PD mouse model, STAT1-mediated IFNγ signaling induced nigrostriatal degeneration [92]. Importantly, preventing STAT1 phosphorylation by administration of ruxolitinib, blocked STAT1 signaling and CXCL10 overexpression in SPG11 iMGL. Moreover, ruxolitinib was capable of rescuing increased neuronal cell death induced by SPG11 iMGL after IFNγ treatment. This implies that proinflammatory factors specifically released from SPG11 iMGL via the IFNγ/ STAT1 signaling pathway are toxic to SPG11 neurons, which are known to exhibit an increased vulnerability and higher cell death rates [77]. Ruxolitinib is a JAK 1/2 inhibitor that has been approved for myelofibrosis and is a candidate for the treatment of several inflammatory or autoimmune diseases [91, 97, 105]. However, the precise mechanism of how *SPG11* loss of function leads to an increased STAT1 activation even after 24 h of IFNγ stimulation remains to be deciphered.

CXCL10 is a chemokine upregulated upon IFNγ/ STAT1 signaling in microglia and other cell types [24, 69]. Consistent with this, IFNγ stimulated SPG11 iMGL exhibited increased expression of CXCL10, but also of the closely related CXCL9 and CXCL11. The latter has been associated not only with neuroinflammation but also with neurodegeneration [49, 107]. In MS, for instance, pathogenic CXCR3-expressing T cells invade the CNS, while activated macrophages and astrocytes predominantly express CXCL9 and CXCL10 in demyelinating lesions [3, 88]. In AD *postmortem* brain tissue, CXCL10 was increased in reactive astrocytes, while CXCR3 was constitutively expressed in a subpopulation of neurons, suggesting that CXCL10 binding could lead to neuronal dysfunction and apoptosis [20, 65, 94, 104].

As an IFNγ-induced chemokine, CXCL10 recruits predominantly CD8^+^ T cells into the CNS [21, 48, 95]. We observed infiltration of CD8^+^ T cells in severely affected brain regions of SPG11 *postmortem* brains, similar to observations in the SPG11^−/−^ mouse model [41]. Of note, infiltrating T cells are a major source of IFNγ in the CNS [43]. Whether microglia are a relevant source of IFNγ remains controversial [9, 22, 61]. In light of our findings and as summarized in Fig. 7, excessive invasion of T cells may contribute to a hyperactivated state of CNS resident myeloid cells via IFNγ signaling, which is further potentiated by cell-intrinsic spatacsin loss of function. In addition, excessive secretion of chemokines such as CXCL10 could further recruit reactive T cells to the CNS, initiating a vicious circle of neurodegeneration and microglia/ T-cell activation. It has been reported that IFNγ-dependent activation of microglia is more pronounced in regions of neurodegeneration than in regions with intact neuron–microglia interplay [66]. Both genetic ablation of T cells and application of T-cell-directed immunomodulators attenuated neurodegeneration in the Spg11^−/−^-KO mouse model, but data regarding their effects on microglial activation are not available [73, 89]. Our findings thus prompt the additional evaluation of emerging compounds specifically targeting the myeloid cell population in SPG11–HSP. Microglia may indeed act upstream of T-cell activation, since ablation of microglia prevented T-cell invasion and neurodegeneration in a mouse model of tauopathy which was dependent on IFNγ signaling [19].

In summary, we here describe for the first time a microglia-associated inflammatory phenotype in SPG11–HSP patients and uncover a STAT1-dependent interplay between *SPG11* and IFNγ driving hyperactivation in *SPG11*-deficient myeloid cells that has the capacity to contribute to neuronal degeneration.

### Supplementary Information

Below is the link to the electronic supplementary material.

Supplementary file1 (DOCX 12526 KB)

## Data Availability

Additional data, material, and protocols are provided upon reasonable request to the corresponding author.
