# Supplementary material for: Neuroinflammatory disease signatures in SPG11-related hereditary spastic paraplegia patients
Source: Acta Neuropathol. 2024 Feb 2;147(1):28. doi: 10.1007/s00401-023-02675-w (PMC10837238; doi:10.1007/s00401-023-02675-w)
Supplement: Supplementary file 1 — Supplementary file1 (DOCX 12526 KB) [file 401_2023_2675_MOESM1_ESM.docx]

**Supplementary figures and tables**

**Neuroinflammatory disease signatures in *SPG11*-related hereditary spastic paraplegia patients**

Laura Krumm(1), Tatyana Pozner(1), Naime Zagha(1), Roland Coras(2), Philipp Arnold(3), Thanos Tsaktanis(4), Kathryn Scherpelz(5), Marie Y. Davis(6,7), Johanna Kaindl(1), Iris Stolzer(8), Patrick Süß(4), Mukhran Khundadze(9), Christian A. Hübner(9,10), Markus J. Riemenschneider(11), Jonathan Baets(12,13,14), Claudia Günther(8,15), Suman Jayadev(6,16,17), Veit Rothhammer(4), Florian Krach(1), Jürgen Winkler(18,19), Beate Winner(1,18)***** and Martin Regensburger(1,15,18,19)*****

**Author affiliations:**

1. Department of Stem Cell Biology, Friedrich-Alexander-Universität (FAU) Erlangen-Nürnberg, Erlangen, Germany
2. Department of Neuropathology, FAU Erlangen-Nürnberg, Erlangen, Germany
3. Institute of Functional and Clinical Anatomy, FAU Erlangen-Nürnberg, Erlangen, Germany
4. Department of Neurology, University Hospital Erlangen, FAU Erlangen-Nürnberg, Erlangen, Germany
5. Division of Neuropathology, Department of Laboratory Medicine and Pathology, University of Washington, Seattle, Washington (WA), USA
6. Department of Neurology University of Washington Medical Center, Seattle, WA, USA
7. VA Puget Sound Healthcare System, Seattle, WA, USA
8. Department of Medicine 1, University Hospital Erlangen, FAU Erlangen-Nürnberg, Erlangen, Germany
9. Institute of Human Genetics, Jena University Hospital, Friedrich-Schiller-University Jena, Jena, Germany
10. Center for Rare Diseases, Jena University Hospital, Friedrich Schiller University Jena, Jena, Germany
11. Department of Neuropathology, Regensburg University Hospital, Regensburg, Germany
12. Translational Neurosciences, Faculty of Medicine and Health Sciences, University of Antwerp, Antwerp, Belgium
13. Laboratory of Neuromuscular Pathology, Institute Born-Bunge, University of Antwerp, Antwerp, Belgium
14. Neuromuscular Reference Centre, Department of Neurology, Antwerp University Hospital, Antwerp, Belgium
15. Deutsches Zentrum Immuntherapie (DZI), University Hospital Erlangen, Erlangen, Germany
16. Institute for Stem Cell and Regenerative Medicine, University of Washington, Seattle, WA, USA
17. Division of Medical Genetics, University of Washington, Seattle, WA, USA
18. Center for Rare Diseases Erlangen (ZSEER), University Hospital Erlangen, FAU Erlangen-Nürnberg, Erlangen, Germany
19. Department of Molecular Neurology, FAU Erlangen-Nürnberg, Erlangen, Germany

* equal contribution

**
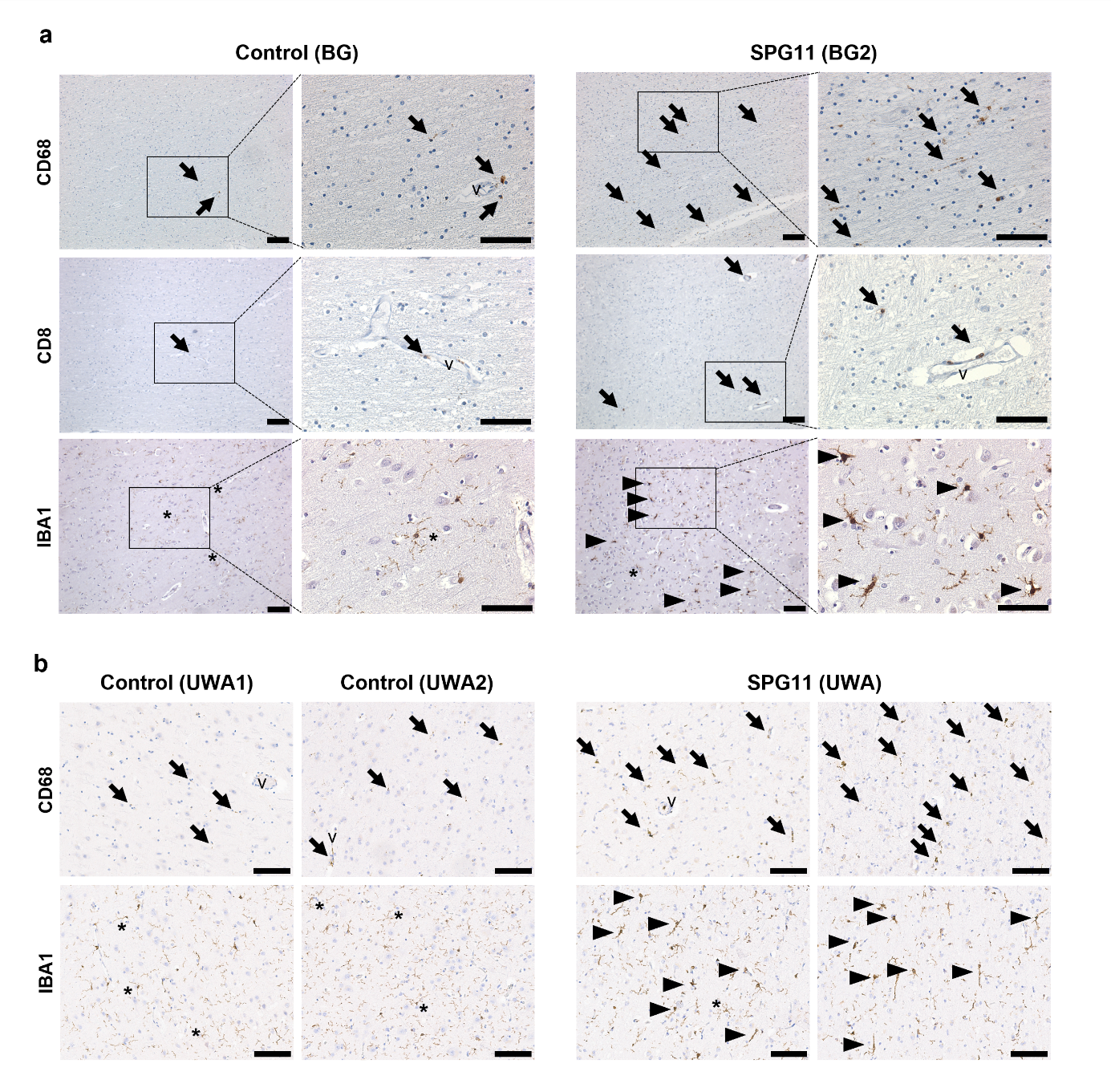
**

**Online Resource Fig. 1. Related to Fig. 2**

**a** The SPG11 (BG2) *postmortem* case exhibits an increase in CD68^+^ myeloid cells, infiltration of CD8^+^ cells (arrows) and accumulation of IBA1^+^ microglia with amoeboid reactive-like morphology (arrowheads) in the frontal lobe compared to a corresponding control (BG). IBA1^+^ microglia with ramified morphology are indicated by * **b** Increase in CD68^+^ myeloid cells (arrows) and amoeboid reactive-like IBA1^+^ microglia (arrowheads) in the frontal lobe of the SPG11 (UWA) *postmortem* case compared to corresponding controls (UWA1 and UWA2). IBA1^+^ microglia with ramified morphology are indicated by *. Scale bars 100 µm. *v* vasculature.


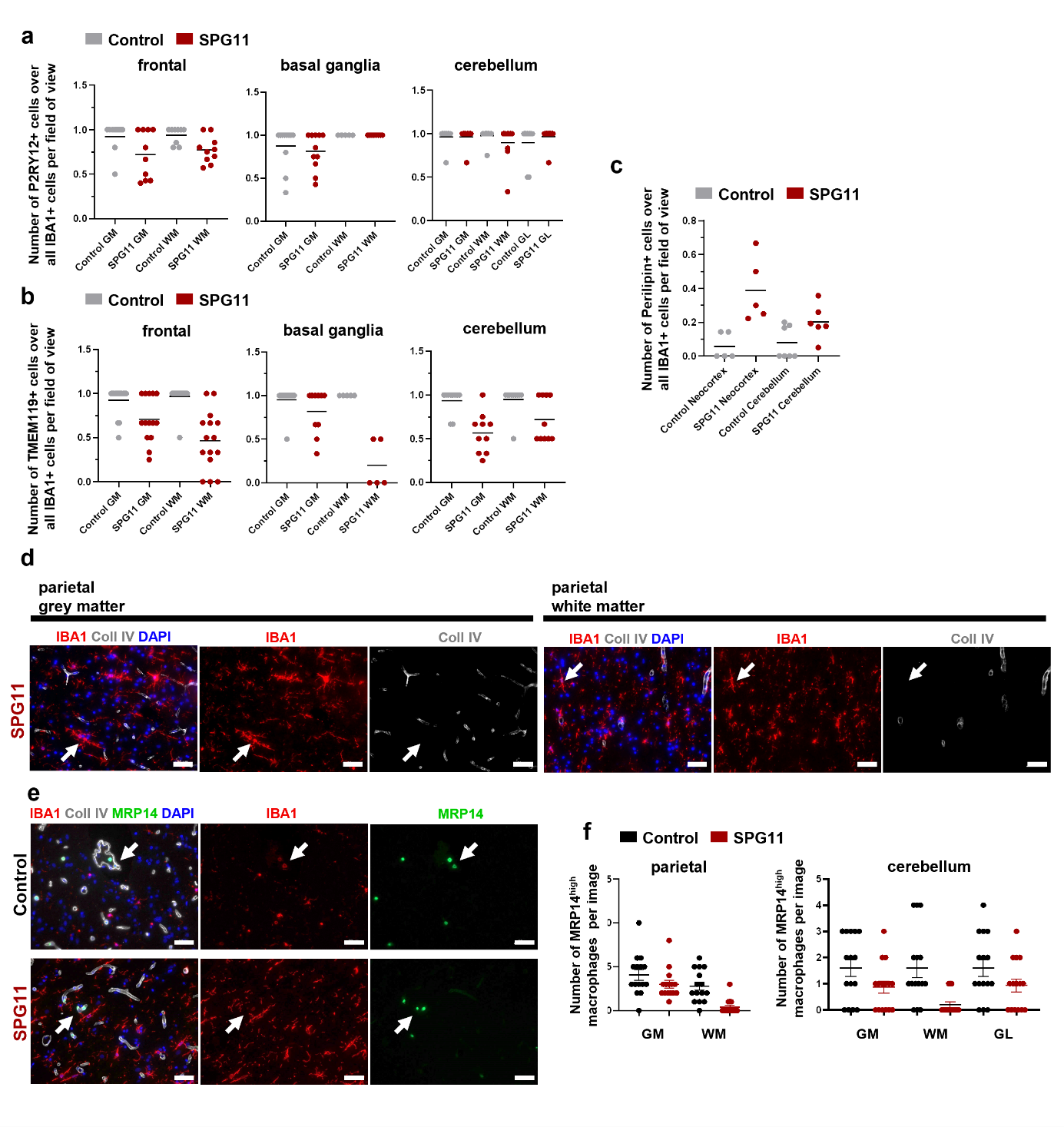


**Online Resource Fig. 2. Related to Fig. 2**

**a+b** Ratio of P2RY12^+^ (a) and TMEM119^+^ (b) over all IBA1^+^ microglia. Each dot represents cell counts from one randomly selected field of view (0.014 mm^2^). *n* = 5-10. **c** Number of perilipin^+^ cells over all IBA1^+^ microglia. Each dot represents results from one randomly selected field of view (0.014 mm^2^). *n* = 5. **d** In the SPG11 parietal lobe, IBA1^+^ microglia (arrow) are evenly distributed in the parenchyma of the white matter and grey matter and do not cluster to vessels (labeled by Coll IV).

*GM* grey matter; *WM* white matter; *GL* granular cell layer, *Coll IV* collagen IV.

**
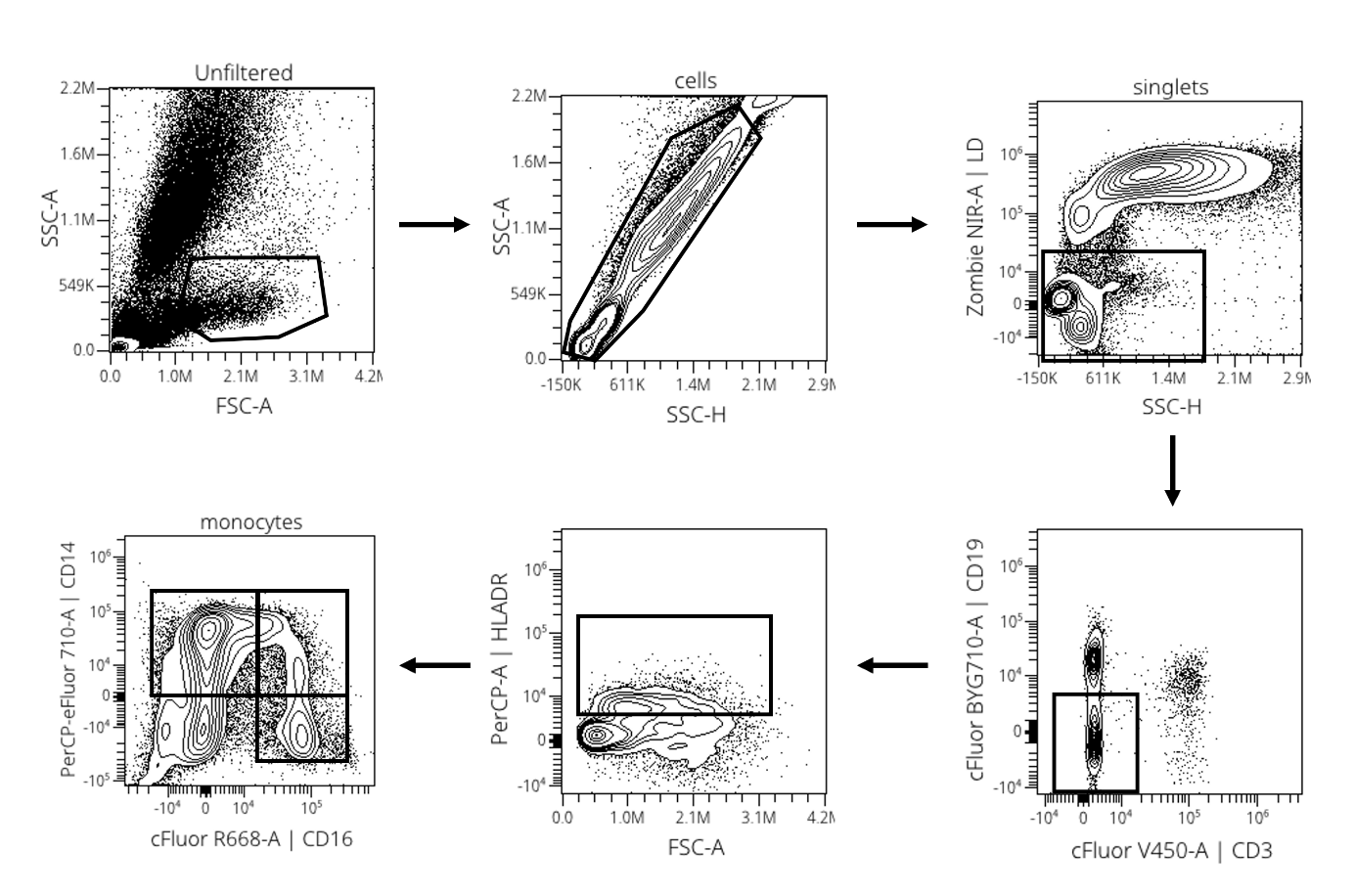
**

**Online Resource Fig. 3. Related to Fig. 3**

Flow cytometric gating strategy of PBMCs to analyze the monocyte subpopulations. The gating was adjusted accordingly [1–3]. Following FSC-A/SSC-A discrimination, a gate was created around the cluster of monocytes using SSC vs. SSC to remove most of the debris, neutrophils, and lymphocytes. Then, using single cell and live gating (LD), T-cells and B-cells were excluded (CD19^-^, CD3^-^), followed by the exclusion of NK-cells using CD56 (not shown). HLA-DR positive monocytes were subsequently analyzed on a CD14 vs. CD16 graph. The proportion of monocytes is determined based on CD14^++^/CD16^-^  (referred to as classical monocytes), CD14^++^/CD16^+^ (intermediate monocytes), and CD14^low/+^/CD16^++^ (non-classical monocytes). *CD* cluster of differentiation, *LD* Live/Dead, *NK* natural killer cells.


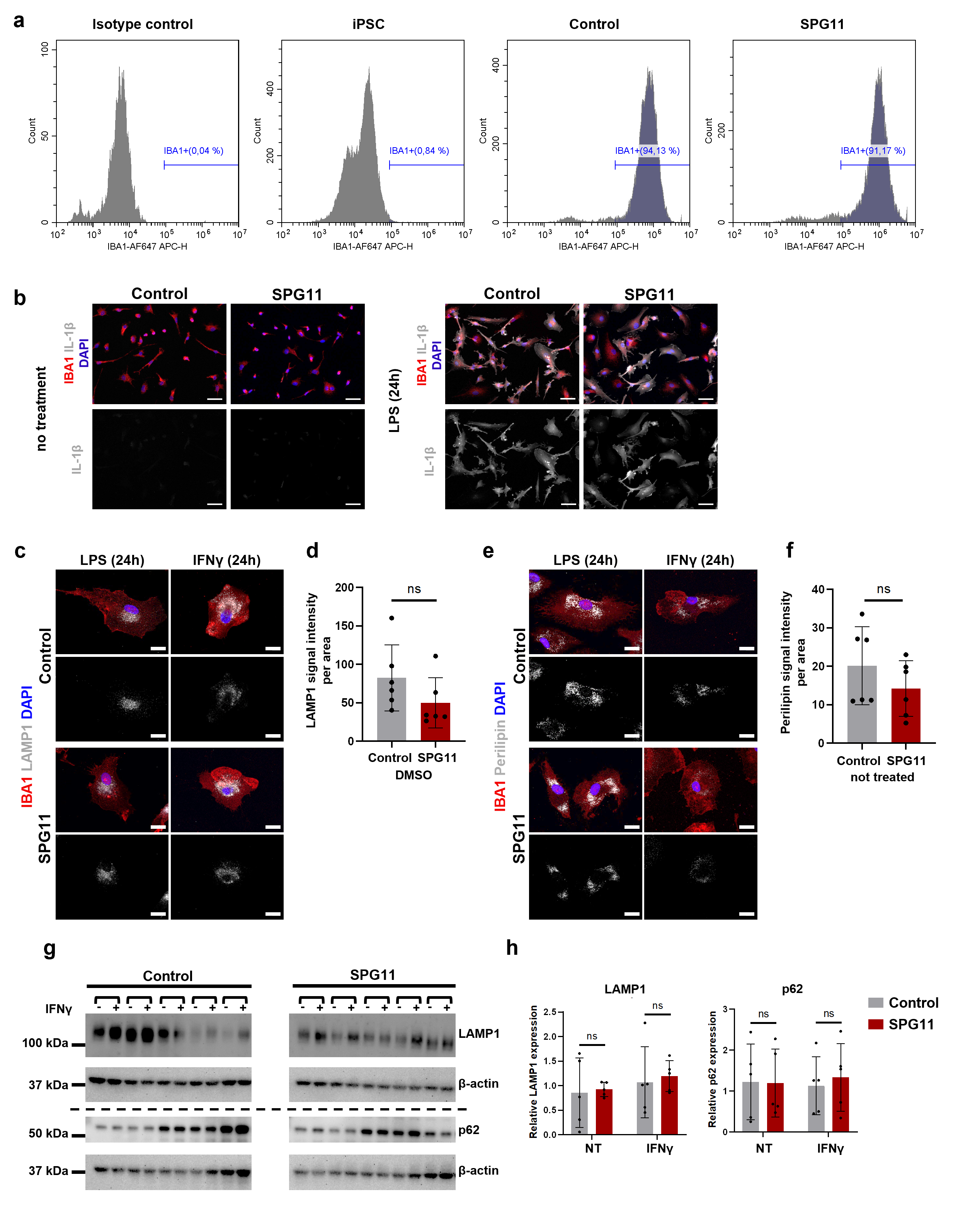


**Online Resource Fig. 4. Related to Fig. 4**

**a** Gating strategy for flow cytometry analysis of IBA1 expression of iMGL. Histograms depicting number of cells (count, y-axis) vs. fluorescence intensity of anti-IBA1 stained and isotype stained iMGL (control and SPG11). iPSCs stained for IBA1 served as an additional negative control. The gating, which was used for analysis of IBA1-positive cells in the respective analysis is indicated in the histograms. **b** Immunofluorescence of control and SPG11 iMGL for IBA1 and IL-1β with and without LPS treatment (100 ng/ml for 24 h). Scale bar: 50 µm. **c** Immunofluorescence of control and SPG11 iMGL for IBA1 and LAMP1. LPS: 100 ng/µl for 24 h. IFNγ: 10 ng/µl for 24 h. Scale bar: 10 µm. **d** Quantification of LAMP1 signal intensity in control (grey) and SPG11 (red) iMGL. Each dot represents the mean of cells within ten random fields of view); bars represent means ± SD. **e** Immunofluorescence of iMGL for IBA1 and perilipin. LPS: 100 ng/µl for 24 h. IFNγ: 10 ng/µl for 24 h. Scale bar: 10 µm. **f** Perilipin fluorescence signal intensity of control (grey) and SPG11 (red) iMGL. Each dot represents mean intensity of one cell line (ten images per line); bars indicate means ± SD. *n* = 6. *P* > 0.05, according to an unpaired *t*-test. **g** Immunoblotting of control and SPG11 iMGL for LAMP1 and p62. β-actin was used as a loading control. Brackets indicate the untreated vs. IFNγ treated (10 ng/µl for 24 h) condition for each cell line. The dashed line separates different membranes. **h** Densitometric quantification of Western blots normalized to β-actin. Data are presented as mean ± SD. *n* = 5. *ns* *P* > 0.05, according to a two-way ANOVA with Bonferroni’s multiple comparison test.

**
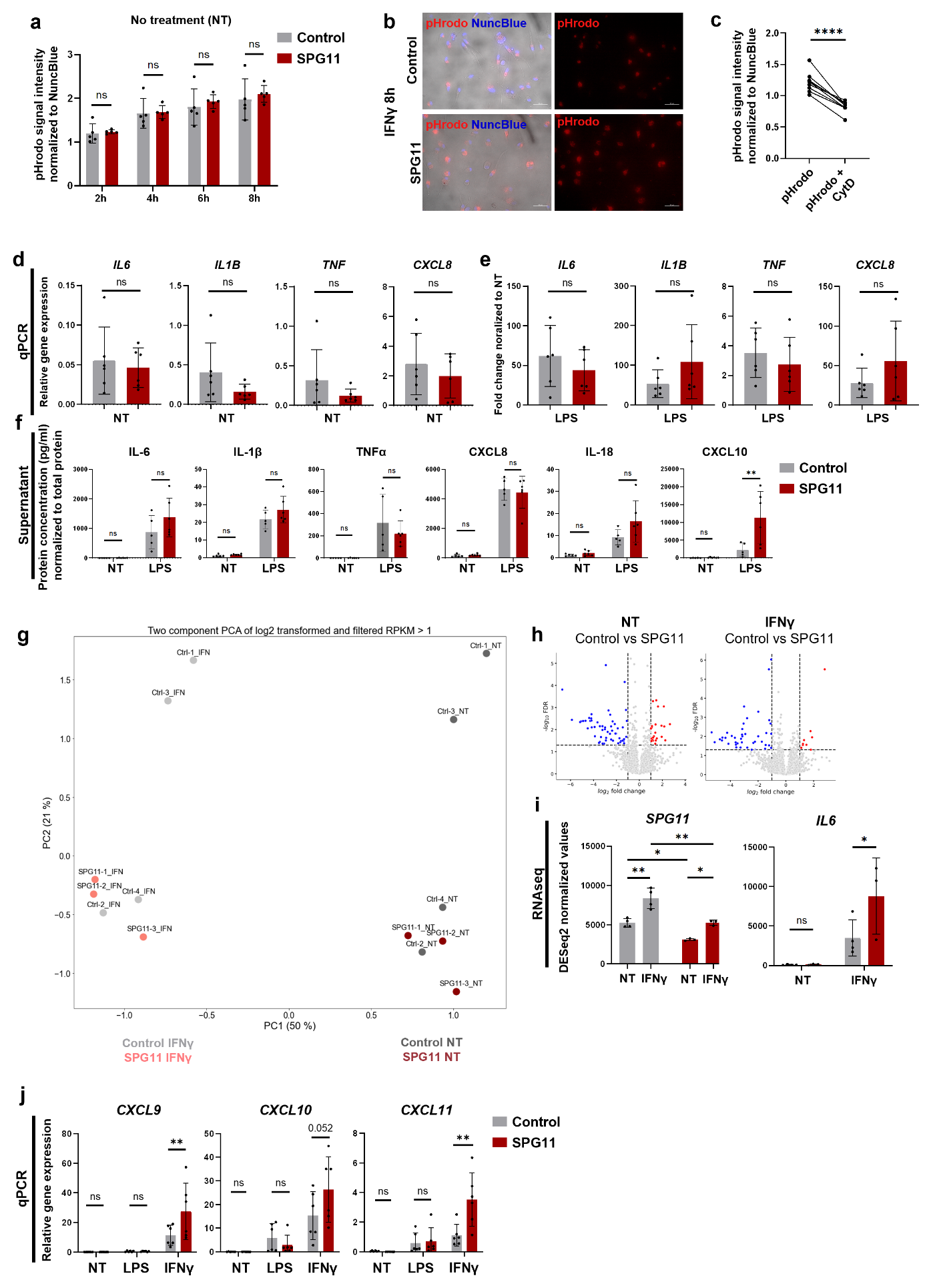
**

**Online Resource Fig. 5. Related to Fig. 5**

**a** Signal intensity of bacterial pHrodo particles in non-treated control (grey) and SPG11 (red) iMGL was normalized to cell density measured by NuncBlue. Fluorescence signal was measured 2 h, 4 h, 6 h and 8 h after adding bacterial particles. *n* = 5. Data presented as mean ± SD. *P* value, according to a two-way ANOVA with Bonferroni’s multiple comparison test. **b** Signal intensity of pHrodo particles in non-treated and CytD treated iMGL, normalized to cell density measured by NuncBlue. *n* = 5. Control and SPG11 iMGL were combined. *P* < 0.0001 according to an unpaired t-test. **c** Representative images depicting NuncBlue and pHrodo fluorescence signal of IFNγ treated (10 ng/ml for 24 h) control and SPG11 iMGL, 8h after adding bacterial particles. Scale bar: 50 µm. **d** Relative gene expression of non-treated control (grey) and SPG11 (red) iMGL. *n* = 6. Data presented as mean ± SD. *P* value according to a non-parametric Mann-Whitney-U test. **e** Relative gene expression of LPS treated control (grey) and SPG11 (red) iMGL normalized to non-treated values of each line. *n* = 6. Data presented as mean ± SD. *P* value, according to a non-parametric Mann-Whitney-U test. **f** Protein concentrations in the supernatant of non-treated and LPS treated control (grey) and SPG11 (red) iMGL normalized to total protein. *n*(control) = 5; *n*(SPG11) = 6. Data presented as mean ± SD. *P* value according to two-way ANOVA with Bonferroni’s multiple comparison test. **g** PCA analysis presenting PC1 and PC2 of all RPKM values in the dataset with a mean RPKM ≥ 1 across all samples. *n*(control) = 4, *n*(SPG11) = 3. NT: controls in light grey, SPG11 in light red. IFNγ treated: controls in dark grey, SPG11 in dark red. **h** Volcano plot illustrating log_2_(fold change) and negative log_10_(adjusted *P* value) from DESeq2 output comparing IFNγ treated iMGL with iMGL that were not subject to any treatment. Only genes with RPKM values in our dataset with a mean RPKM ≥ 1 across all samples were considered. red: adjusted *P* value < 0.05, log_2_(fold change) > 1; blue: adjusted p value ≤ 0.05, log_2_(fold change) < 1. **i** DESeq2 normalized gene expression values of untreated and IFNγ treated iMGL (control in grey and SPG11 in red). DESeq output filtered for adjusted *P* value < 0.05 and log2(fold change) > 1. *n*(SPG11) = 3, *n*(control) = 4. * P < 0.05, according to two-way ANOVA with Bonferroni’s multiple comparison test. **j** Relative gene expression in control (grey) and SPG11 (red) iMGL treated with IFNγ or LPS (100 ng/ml for 24 h). *n* = 6. Data presented as mean ± SD. *P* value according to two-way ANOVA with Bonferroni’s multiple comparison test. *CytD* CytochalasinD; *NT* not treated; ns *P* > 0.05, * *P* < 0.05, ** *P* < 0.01.

**
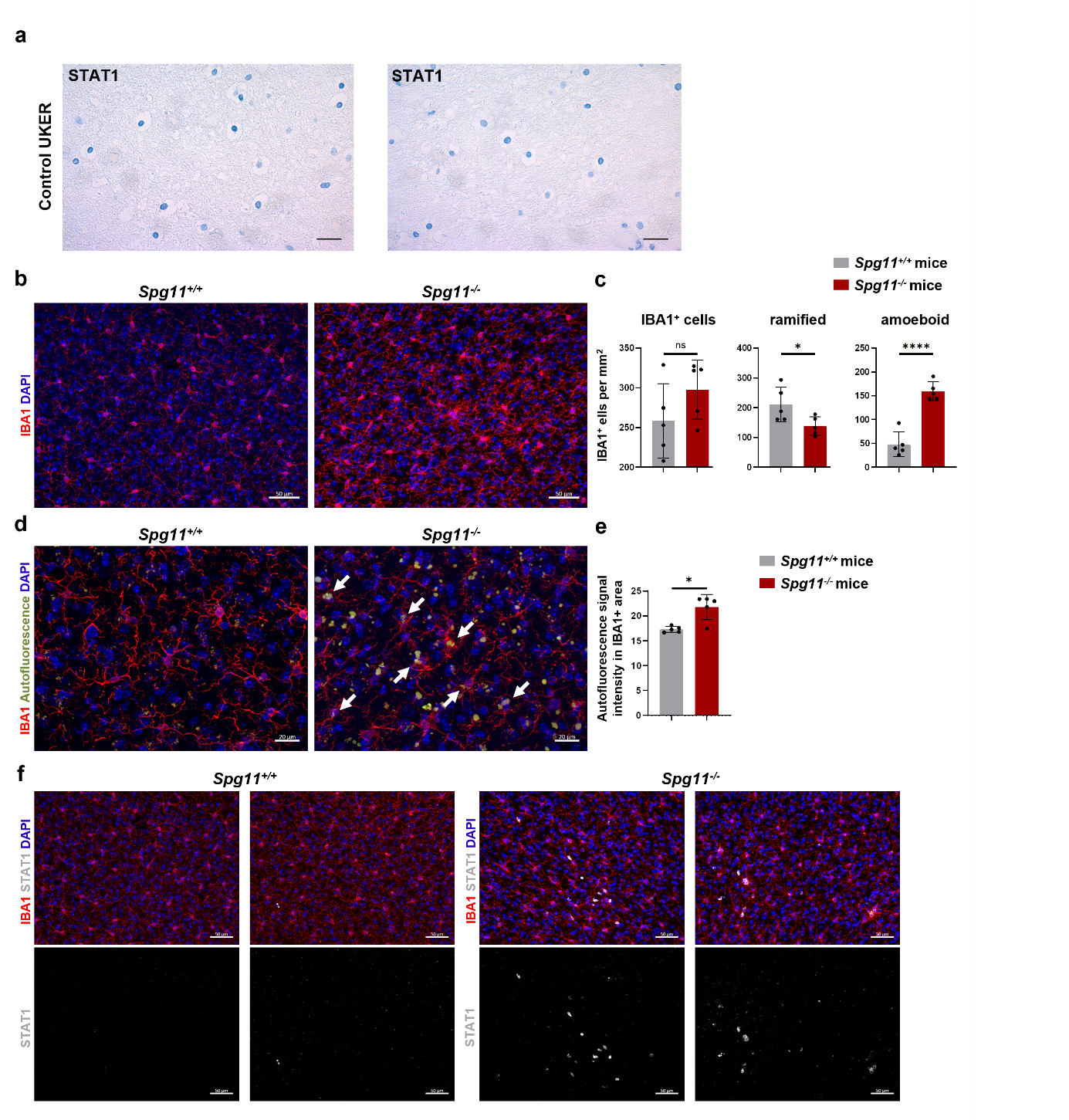
**

**Online Resource Fig. 6. Related to Fig. 5**

**a** Two representative images of STAT1 stained control UKER postmortem parietal lobe. Scale bar 20 µm. **b** Representative immunofluorescence images of IBA1 and DAPI of the frontal lobe of *Spg11^+/+^* and *Spg11^-/-^* mice. Scale bar: 50 µm. **c** Quantification of IBA1+ cells with ramified homeostatic-like or amoeboid reactive-like morphology. *n*(*Spg11^+/+^*) = 5; *n*(*Spg11^-/-^*) = 5. Each dot represents the mean of cells within five random fields of view. Data presented as mean ± SD. *P* value according to a non-parametric Mann-Whitney-U test. **d** Representative immunofluorescence images showing autofluorescence material in IBA1^+^ microglia in the cortex of Spg11^-/-^ mice indicated by arrows. Scale bar: 20 µm. **e** Quantification of the autofluorescence signal intensity within the IBA1^+^ area. *n*(*Spg11^+/+^*) = 5; *n*(*Spg11^-/-^*) = 5. Each dot represents the mean of cells within five random fields of view. Data presented as mean ± SD. *P* value according to a non-parametric Mann-Whitney-U test. **f** Representative immunofluorescence images of IBA1, STAT1 and DAPI in the frontal lobe of *Spg11^+/+^* and *Spg11^-/-^* mice. Scale bar: 50 µm.

**
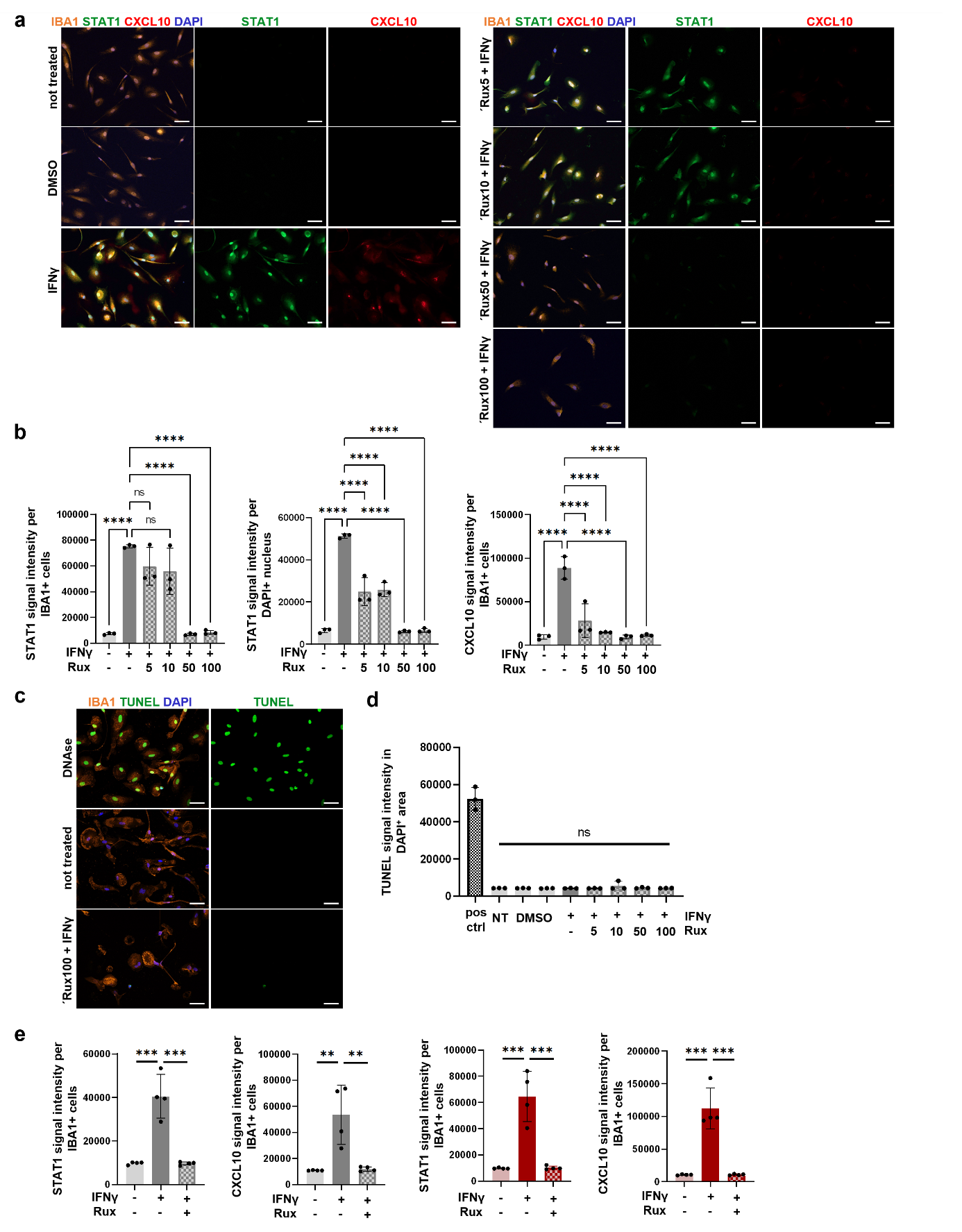
***ns* *P* > 0.05; * *P* < 0.05; **** *P* < 0.0001

**Online Resource Fig. 7. Related to Fig. 6**

**a** Representative immunofluorescence for IBA1, STAT1 and CXCL10 in control iMGL treated with IFNγ (10 ng/ml for 24 h) and Ruxolitinib (Rux, 5-100 µM, 24 h prior IFNγ). DMSO control was included. **b** Quantification of STAT1 signal intensity within IBA1^+^ iMGL and DAPI^+^ nuclei and CXCL10 signal intensity within IBA1^+^ iMGL. Each dot represents the mean of cells one random field of view (0.15 mm^2^). **c** To visualize apoptotic cells, a TUNEL assay followed by immunofluorescence staining for IBA1 was performed. Representative images of control iMGL. DNAse was used as a positive control. **d** Quantification of TUNEL signal intensity within DAPI^+^ nuclei. Each dot represents the mean of one random field of view (0.15 mm^2^). **e** Quantification of mean fluorescence intensity of STAT1 and CXCL10 within IBA1^+^ control (*n* = 4) and SPG11 (*n* = 4) iMGL. Each dot represents the mean of cells within five random fields of view.

Scale bar: 50 µm. Bars represent means ± SD. *P* value according to one-way ANOVA with Bonferroni’s multiple comparison test. ns *P* > 0.05, ** *P* < 0.01, *** *P* < 0.001, **** *P* < 0.0001. *NT* not treated *Rux* Ruxolitinib. *pos ctrl* positive control.


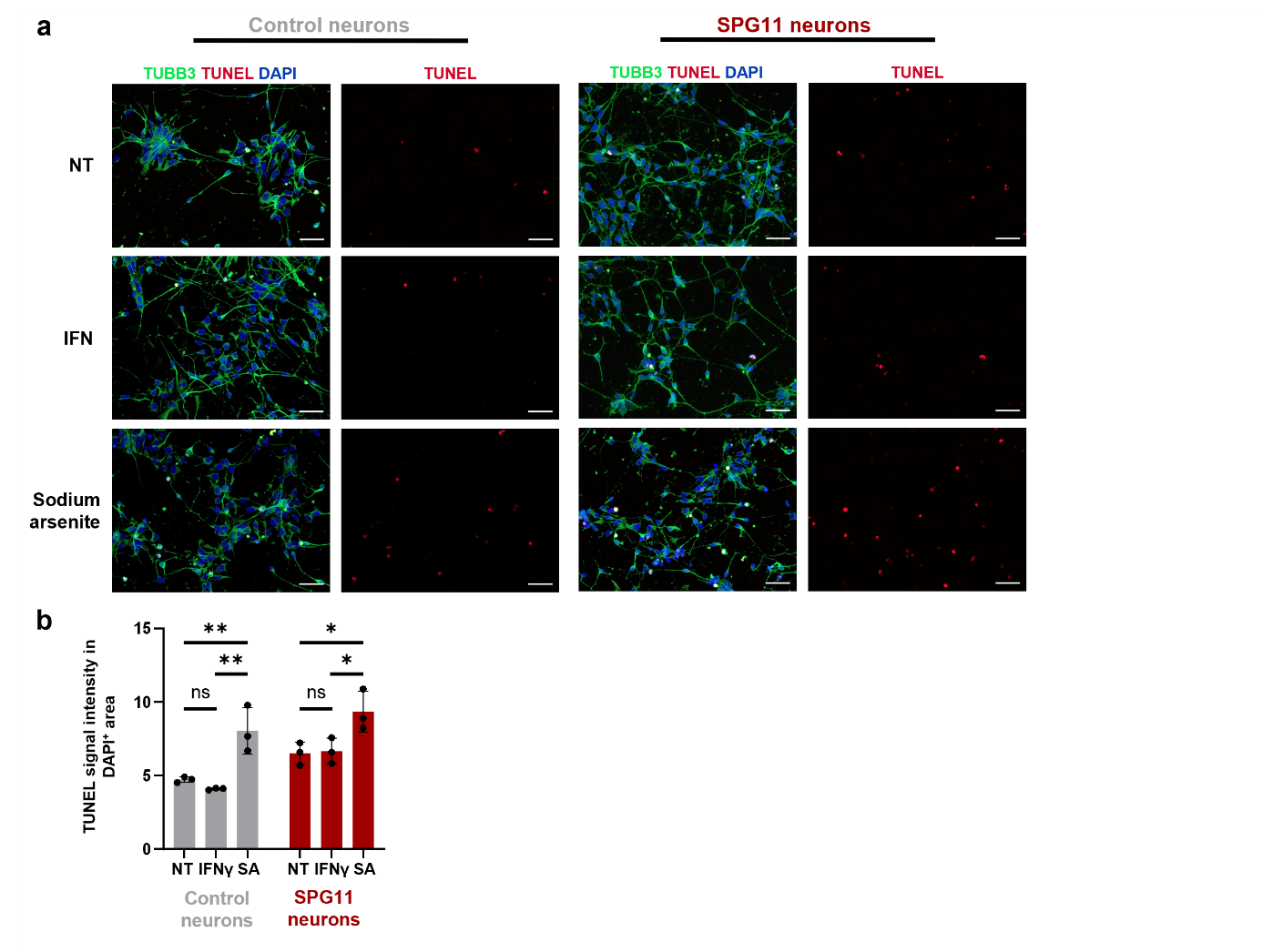


**Online Resource Fig. 8. Related to Fig. 6**

**a** Representative immunofluorescence images of control and SPG11 neurons stained for TUBB3 and DAPI. Apoptotic cells were visualized by TUNEL assay. Neurons were treated with IFNγ (10 ng/ml for 24h) and sodium arsenite (5 nM for 24h) as a positive control. Scale bar: 50 µm. b Quantification of apoptotic cells by TUNEL signal intensity within the DAPI^+^ nuclei. *n*(control)=1 and n(SPG11)=1. Each dot represents the mean of cells within five random fields of view. Bars represent means ± SD. *P* value according to two-way ANOVA with Bonferroni’s multiple comparison test*. ns* *P* > 0.05; * *P* < 0.05; ** *P* < 0.01. Column factor comparing control vs SPG11: *P*=0.0016.

**Online Resource** **Table 1** SPG11-HSP patient cohort and respective controls of the PBMC analysis.

| **Parameter**  **(mean ± SD)** | **SPG11-HSP**  **n=8** | **Controls**  **n=38** | ***P* value** |
| --- | --- | --- | --- |
| **Age (y)** | 35.00 ±11.78 | 45.68 ±17.43 | 0.11 |
| **Sex (male:female)** | 1:7 | 11:27 | 0.69 |

*SD* standard deviation, *y* years.

**Online Resource** **Table 2** SPG11-HSP patient cohort and respective controls of the serum analysis.

| **Parameter**  **(mean ± SD)** | **SPG11-HSP**  **n=13** | **Controls**  **n=20** | ***P* value** |
| --- | --- | --- | --- |
| **Age (y)** | 32.34 ±11.98 | 39.05 ±13.70 | 0.28 |
| **Sex (male:female)** | 6:7 | 8:12 | 0.73 |
| **Spastic paraplegia rating score (0-52)** | 30.38 ±11.22 | - | - |

*SD* standard deviation, *y* years.

**Online Resource** **Table 3** Baseline characterization of SPG11-HSP patients and Control subjects from whom iPSC lines were derived.

|  | **SPG11-1** | **SPG11-2** | **SPG11-3** | **Control-1** | **Control-2** | **Control-3** | **Control-4** | **SPG11-HA** |
| --- | --- | --- | --- | --- | --- | --- | --- | --- |
| *SPG11* variants | Exon 16: c.3036C>A Exon 30: c.5798delC | Exon 16: c.3036C>A Exon 30: c.5798delC | Exon 2: c.267G>A Intron 6: c.1457-2A>G | - | - | - | - | C-terminal HA tag  (bi-allelic) |
| Age at onset /age at examination | 24 / 46 | 20 / 40 | 31 / 50 | - / 45 | - / 66 | - / 52 | - / - | - / - |
| Sex | female | female | female | female | female | female | female | female |
| Age at biopsy | 40 | 34 | 43 | 45 | 65 | 52 | neonatal | neonatal |
| Spastic paraplegia rating score (0-52) | 44 | 37 | 36 | - | - | - | - | - |
| Cognitive impairment | + | + | + | - | - | - | - | - |
| MRI abnormalities | Cortical atrophy, white matter lesions, thinning of the corpus callosum | | | - | - | - | - | - |
| iPSC clones | SPG11-1a/b | SPG11-2a/b | SPG1-3a/b | Ctrl-1a/b | Ctrl-2a/b | Ctrl-3a/b | Ctrl-4 | SPG11-HA |
| Full identifier of individual | UKERi6O6-R | UKERi4AA-S | UKERiK22-S | UKERi33Q-S1 | UKERi82A-S1 | UKERi7MN-S | TMOi001-A | TMOi001-A-4 |

*iPSC* induced pluripotent stem cells.

**Online Resource Table 4** Antibodies.

| **Antibody** | | **Manufacturer** | **Catalog number** | **Working dilution** |
| --- | --- | --- | --- | --- |
| Anti-goat AF 546 | donkey | Thermo Fisher | A11056 | IHC/ICC: 1:500 |
| Anti-goat AF 647 | donkey | Thermo Fisher | A21447 | IHC/ICC: 1:500 |
| Anti-mouse AF 488 | donkey | Thermo Fisher | A32766 | IHC/ICC: 1:500 |
| Anti-mouse AF 546 | donkey | Thermo Fisher | A10036 | ICC: 1:500 |
| Anti-rabbit AF 488 | donkey | Thermo Fisher | A32790 | IHC/ICC: 1:500 |
| Anti-rabbit AF 546 | donkey | Thermo Fisher | A10040 | IHC/ICC: 1:500 |
| Anti-rabbit AF 647 | donkey | Thermo Fisher | A31573 | FC: 1:200  IHC/ICC: 1:500 |
| Anti-chicken AF 488 | donkey | Thermo Fisher | A78948 | ICC: 1:500 |
| Collagen IV | goat | Sigma-Aldrich | AB769 | IHC: 1:100 |
| CXCL10 | goat | R&D Systems | AF-266 | ICC: 1:200 |
| HA | mouse | Abcam | Ab18181 | WB: 1:1000 |
| IBA1 | mouse | Sigma-Aldrich | MABN92 | ICC: 1:200 |
|  | rabbit | Wako | 019-19741 | IHC: 1:300  ICC: 1:500  FC: 1:50 |
|  | goat | Abcam | ab5076 | IHC: 1:500 |
| IL-1β | goat | R&D Systems | AF-201-NA | ICC: 1:300 |
| LAMP1 | rabbit | Abcam | Ab24170 | ICC: 1:200  WB: 1:3500 |
| P2RY12 | rabbit | Sigma-Aldrich | HPA014518 | IHC: 1:200 |
| P62 | rabbit | Millipore | MAPC32 | WB: 1:3500 |
| Perilipin | mouse | Santa Cruz | Sc-390169 | IHC: 1:100 |
| Perilipin 2 | rabbit | Sigma-Aldrich | HPA016607 | ICC: 1:200 |
| P-STAT1 | rabbit | Cell Signaling | 9167 | WB: 1:1000 |
| STAT1 | rabbit | Cell Signaling | 9172 | WB: 1:1000 |
|  | rabbit | Cell Signaling | 14994 | ICC: 1:200  IHC: 1:400 |
| TMEM119 | rabbit | Abcam | ab185333 | IHC: 1:250 |
| TUBB3 | chicken | Millipore | AB9354 | ICC: 1:300 |
| β-actin | mouse | SIGMA | A1978 | WB: 1:5000 |

**Online Resource Table 5** qPCR Primers.

| **Target mRNA** | **Forward primer 5’-3’** | **Reverse Primer 5’-3’** |
| --- | --- | --- |
| *SPG11* | CAAGGACCTAAGGGCGTAGAT | TTCAATGATGATAGCTGGGCTTT |
| *IL1B* | TCCAGGGACAGGATATGGAG | CCCAAGGCCACAGGTATTT |
| *IL6* | AGACAGCCACTCACCTCTTCAG | TTCTGCCAGTGCCTCTTTGCTG |
| *TNF* | TCAGCCTCTTCTCCTTCCTG | GCCAGAGGGCTGATTAGAGA |
| *CXCL8* | AGCACTCCTTGGCAAAACTG | CGGAAGGAACCATCTCACTG |
| *CXCL9* | CCAGTAGTGAGAAAGGGTCGC | AGGGCTTGGGGCAATTGTT |
| *CXCL10* | GTGGCATTCAAGGAGTACCTC | TGATGGCCTTCGATTCTGGAT |
| *CXCL11* | GACGCTGTCTTTGCATAGGC | GGATTTAGGCATCGTTGTCCTTT |
| *GAPDH* | GTCGGAGTCAACGGATTTG | TGGGTGGAATCATATTGGAAC |
| *HPRT* | CCTGGCGTCGTGATTAGTG | TCCCATCTCCTTCATCACATC |

# **Supplementary references**

1. Abeles RD, McPhail MJ, Sowter D, Antoniades CG, Vergis N, Vijay GKM, Xystrakis E, Khamri W, Shawcross DL, Ma Y, Wendon JA, Vergani D (2012) CD14, CD16 and HLA-DR reliably identifies human monocytes and their subsets in the context of pathologically reduced HLA-DR expression by CD14(hi) /CD16(neg) monocytes: Expansion of CD14(hi) /CD16(pos) and contraction of CD14(lo) /CD16(pos) monocytes in acute liver failure. Cytometry A 81:823–834. doi: 10.1002/CYTO.A.22104

2. Maecker HT, McCoy JP, Nussenblatt R (2012) Standardizing immunophenotyping for the Human Immunology Project. Nat Rev Immunol 12:191–200. doi: 10.1038/NRI3158

3. Zungsontiporn N, Tello RR, Zhang G, Mitchell BI, Budoff M, Kallianpur KJ, Nakamoto BK, Keating SM, Norris PJ, Ndhlovu LC, Souza SA, Shikuma CM, Chow DC (2016) Non-Classical Monocytes and Monocyte Chemoattractant Protein-1 (MCP-1) Correlate with Coronary Artery Calcium Progression in Chronically HIV-1 Infected Adults on Stable Antiretroviral Therapy. PLoS One 11. doi: 10.1371/JOURNAL.PONE.0149143
